# Identification of active and taxonomically diverse 1,4-dioxane degraders in a full-scale activated sludge system by high-sensitivity stable isotope probing

**DOI:** 10.1038/s41396-018-0201-2

**Published:** 2018-06-13

**Authors:** Tomo Aoyagi, Fumiaki Morishita, Yutaka Sugiyama, Daisuke Ichikawa, Daisuke Mayumi, Yoshitomo Kikuchi, Atsushi Ogata, Kenji Muraoka, Hiroshi Habe, Tomoyuki Hori

**Affiliations:** 1 Environmental Management Research Institute, National Institute of Advanced Industrial Science and Technology (AIST), 16-1 Onogawa, Tsukuba, 305-8569 Japan; 2 NIPPON SHOKUBAI Co., Ltd., 14-1 Chidorichou, Kawasaki, 210-0865 Japan; 3 Institute for Geo-Resources and Environment, Geological Survey of Japan, National Institute of Advanced Industrial Science and Technology (AIST), 1-1-1 Higashi, Tsukuba, 305-8567 Japan; 4 Bioproduction Research Institute, National Institute of Advanced Industrial Science and Technology (AIST), 2-17-2-1 Tsukisamu-Higashi, Sapporo, 062-8517 Japan

## Abstract

1,4-Dioxane is one of the most common and persistent artificial pollutants in petrochemical industrial wastewaters and chlorinated solvent groundwater plumes. Despite its possible biological treatment in natural environments, the identity and dynamics of the microorganisms involved are largely unknown. Here, we identified active and diverse 1,4-dioxane-degrading microorganisms from activated sludge by high-sensitivity stable isotope probing of rRNA. By rigorously analyzing 16S rRNA molecules in RNA density fractions of ^13^C-labeled and unlabeled 1,4-dioxane treatments, we discovered 10 significantly ^13^C-incorporating microbial species from the complex microbial community. 16S rRNA expression assays revealed that 9 of the 10 species, including the well-known degrader *Pseudonocardia dioxanivorans*, an ammonia-oxidizing bacterium and phylogenetically novel bacteria, increased their metabolic activities shortly after exposure to 1,4-dioxane. Moreover, high-resolution monitoring showed that, during a single year of operation of the full-scale activated sludge system, the nine identified species exhibited yearly averaged relative abundances of 0.001–1.523%, and yet showed different responses to changes in the 1,4-dioxane removal efficiency. Hence, the co-existence and individually distinct dynamics of various 1,4-dioxane-degrading microorganisms, including hitherto unidentified species, played pivotal roles in the maintenance of the biological system removing the recalcitrant pollutant.

## Introduction

The cyclic ether 1,4-dioxane is generated as a byproduct in petrochemical industrial processes and has been widely utilized as a stabilizer for chlorinated solvents [1–3]. The International Agency for Research on Cancer (IARC) and United States Environmental Protection Agency (US EPA) have indicated its possible carcinogenic effects to humans [4, 5]. Due to its toxicity and high mobility, groundwater contamination by 1,4-dioxane is a serious and significant concern over a large region [6, 7]. For this reason, 1,4-dioxane concentrations in public and ground waters are strictly regulated in Japan at below 0.05 mg l^−1^ and those in the treated wastewater at below 0.5 mg l^−1^.

Physical, chemical and biological treatment technologies have been developed to remove 1,4-dioxane from the contaminated environments [8]. The physical treatments include thermal destruction, air stripping, distillation and carbon adsorption, but their efficacy is limited by the solubility, boiling point and vapor pressure of 1,4-dioxane [9]. Chemical treatments, that is, advanced oxidation processes, can effectively decompose 1,4-dioxane within only a few hours [10, 11]. Biological treatments have attracted increasing attention as naturally occurring processes with low cost and low energy [12], and because they require none of the external special equipment that the other abiotic treatments require.

1,4-Dioxane was initially recognized as a non-biodegradable compound, due to its long persistence in wastewater treatment plants [13]. In fact, this compound has been shown to exhibit a high degree of resistance to microbial degradation due to its C–O–C ether linkage, which features a ring structure and high-energy C–O bonds (360 kJ mol^–1^) [14]. However, recent studies have shown that intrinsic microorganisms capable of degrading 1,4-dioxane are more widely distributed in natural environments than previously expected [15–17]. Moreover, a number of 1,4-dioxane-degrading microorganisms have been isolated as a result of intensive efforts made in the last two decades [12]. It has been reported that 10 isolates grow on 1,4-dioxane as a sole carbon and energy source (1,4-dioxane metabolism) [18], whereas 12 isolates utilize 1,4-dioxane only when an additional growth-supporting carbon source is introduced (1,4-dioxane co-metabolism) [19]. In the case of co-metabolism, however, the microorganisms can grow with the primary substrate but not with the compound co-metabolized [20].

It has been shown that the isolated 1,4-dioxane degraders exhibit high phylogenetic diversity. A few strains in the well-known degrader genera *Pseudonocardia* and *Rhodococcus* have 1,4-dioxane degradation abilities [21]. Tracing 16S ribosomal RNA (rRNA) genes has not been possible, because 1,4-dioxane degradation is a widespread trait in the microbial realm, having been detected in many bacterial, as well as fungal lineages [12, 22, 23]. Instead of a universal bio-signature, functional genes encoding monooxygenase enzymes have been proposed as biomarkers for 1,4-dioxane degradation [24], and subsequent studies attempted to improve the accuracy of these biomarkers [25, 26]. However, although this biomarker approach is effective for monitoring the known microorganisms participating in monooxygenase-driven 1,4-dioxane degradation [27–29], it cannot discover hitherto unknown 1,4-dioxane degraders whose genomes lack the monooxygenase-encoding genes or include unusual types of the genes. In fact, the 1,4-dioxane-degrading *Xanthobacter flavus* strain DT8 has been reported to have a monooxygenase-independent degradation pathway [30]. Due to these limitations, there is still only limited information about the phylogenic identity and diversity of the microorganisms involved in the degradation of 1,4-dioxane in natural and contaminated environments.

A direct method to link the identity and function of hitherto unknown microorganisms is stable isotope probing (SIP) of rDNA [31], and particularly of rRNA [32]. 1,4-Dioxane degraders, that is, the main target of this study, exhibit very low degradation rates and growth rates [3, 33, 34], and therefore would be expected to have low abundance in natural environments. Thus, a substantial improvement in the sensitivity of SIP is needed in order to unambiguously detect small amounts of heavily ^13^C-enriched nucleic acids stemming from ^13^C-labeled 1,4-dioxane degraders. Recently, compared with that of SIP with terminal restriction fragment length polymorphism [35, 36], the detection sensitivity has been 500-fold enhanced by high-throughput Illumina sequencing of isopycnic centrifugation gradients, referred to as high-sensitivity rRNA-SIP [37]. Although further rise in the sensitivity may be possible with advent of new technology in future, this sensitive approach at present would unveil the ecophysiology of low-abundance microorganisms that can actively dissimilate but only marginally assimilate the ^13^C-labeled persistent pollutant.

In this study, high-sensitivity rRNA-SIP was first used to identify 1,4-dioxane degraders in a full-scale activated sludge system that is used to effectively treat high concentrations of 1,4-dioxane in petrochemical industrial wastewaters. In addition, both the identified 1,4-dioxane degraders and the whole microbial communities were monitored at high resolution using an Illumina MiSeq platform [38, 39] over 1-year of operation of a full-scale treatment system in order to better understand their population dynamics under changing environmental conditions.

## Materials and methods

### High-sensitivity SIP of 1,4-dioxane degraders

#### Aerobic incubation of activated sludge microorganisms with ^13^C-labeled 1,4-dioxane

An activated sludge was collected on December 2015 from an aeration tank of a full-scale treatment system of petrochemical industrial wastewaters at NIPPON SHOKUBAI Co., Ltd. (Kawasaki, Japan). Twenty milliliters of the sludge were transferred into a 123-ml glass serum vial, which was then sealed with a butyl rubber septum. The gaseous phase was air and the headspace O_2_ concentrations at hour 0 were 0.191−0.210 atm. Three treatments were carried out as follows: (i) ^13^C treatment: non-autoclaved sludge microorganisms were supplemented with ^13^C-labeled 1,4-dioxane (1,4-dioxane-^13^C_4_, 99 atom % ^13^C; Sigma-Aldrich, Japan). (ii) Unlabeled treatment: non-autoclaved sludge microorganisms with unlabeled 1,4-dioxane (Wako, Japan). (iii) Heat-killed treatment: autoclaved sludge microorganisms with unlabeled 1,4-dioxane. Autoclave sterilization of the sludge microorganisms was performed three times at 121 °C for 1 h to kill the sludge microorganisms completely [40]. Before starting the incubation, the chemical oxygen demand (COD) was determined to be 27 mg l^−1^ and 1,4-dioxane was not detectable because it was almost completely absent in the original sludge sample. ^13^C-labeled and unlabeled 1,4-dioxane were added at final concentrations of 200 mg l^−1^ at the start of the incubations. In all, 200 mg l^−1^ of 1,4-dioxane corresponded to 424 mg l^−1^ of COD. The activated sludge microorganisms were aerobically incubated at 25 °C with agitation at 200 rpm for 8 h in the dark. The pH value was nearly constant at 8.0−8.3 throughout the incubation (data not shown). All treatments were run in triplicate. The sludge samples were stored at –80 °C for subsequent chemical and molecular analyses.

#### Chemical analyses during the incubation of activated sludge microorganisms

The headspace gas and sludge water were collected at hours 0, 2, 4, 6 and 8 from each vial of each set of the ^13^C and unlabeled treatments performed in triplicate. Mixed liquor suspended solid (MLSS), that is, total biomass, of the sludge was 10,100 mg l^–1^ at hour 0. Total CO_2_ and O_2_ in the headspace gas were analyzed by a gas chromatograph (GC-14B; Shimadzu, Japan) equipped with a thermal conductivity detector and a packed column (ShinCarbon ST; Shinwa, Japan) [41]. The carbon isotopic composition of gaseous CO_2_ was measured with a gas chromatograph combustion isotope ratio mass spectrometer (GC-C-IRMS) consisting of a Trace GC Ultra, a GC IsoLink, a ConFlo IV and a DELTA V Plus IRMS system (Thermo Fisher Scientific, MA, USA) [42]. The isotopic data were obtained as δ values, that is, per mil derivation of the ^13^C/^12^C ratio relative to the Vienna Pee Belemnite (VPDB) standard, in the IRMS analysis and converted to atom % by calculating (1 + δ/1000) × 1.124. The isotopic composition of dissolved bicarbonate was calculated as δ_HCO3[−]_ = (δ_CO2_ − ε_HCO3[−]_)/(1 + ε_HCO3[−]_/1000), where ε_HCO3(−)_ represents the constants of carbon isotopic fractionation between gaseous CO_2_ and dissolved bicarbonate (i.e., −7.93‰ at 25 °C) [43]. The concentration of 1,4-dioxane was determined with direct injection of the sludge water by a gas chromatograph (GC-2101plus; Shimadzu) equipped with a flame ionization detector and a dimethylsiloxane column DB-1 (Agilent Technologies, Japan). The column temperature was held at 100 °C for 15 min, then increased to 290 °C (40 °C per min) and held at 290 °C for 20 min. Standard solutions of 1,4-dioxane were prepared at concentrations of 0, 5.8, 29.2 and 92.1 mg l^−1^. The detection limit was determined to be 0.17 mg l^−1^. For the heat-killed treatment, the 1,4-dioxane concentration was determined after an 8-h incubation. The inorganic carbon (IC) concentration from the sludge water was determined with a TOC analyzer (TOC-L; Shimadzu). COD of the sludge water was measured with a COD kit (TNT821; Hach, CO, USA) and a COD analyzer (DRB200; Hach). pH was measured by a compact pH meter (Laquatwin; Horiba, Japan).

#### RNA extraction and density gradient centrifugation

RNA was extracted from 2 ml of each sludge sample from the ^13^C and unlabeled treatments done in triplicate after an 8-h incubation. Total nucleic acids extraction was performed using a direct lysis protocol involving bead beating [44]. Then, RNA was purified by DNA digestion with a DNase (RQ1; Promega, Japan). Total RNA was quantified using a RiboGreen RNA quantification kit (Invitrogen, CA, USA) and a microplate reader (SH-900Lab; Corona, Japan). Five-hundred nanograms of RNA mixed with cesium trifluoroacetate (CsTFA) solution (Wako) was subjected in triplicate to ultra-centrifugation with 128,000 *g* for >60 h at 20 °C [45]. Gradients of density-separated RNAs were fractionated, and the CsTFA buoyant density (BD) of each fraction was determined with a refractometer (AR200; Reichert, NY, USA) [45].

#### Reverse transcription (RT)-PCR, Illumina sequencing

The heaviest (1H), second-heaviest (2H), third-heaviest (3H) and light (L) fractions of RNA with BDs of 1.803–1.808, 1.796–1.800, 1.788–1.793 and 1.769–1.771 g ml^–1^, respectively, were subjected to RT-PCR with a one-step amplification system (Access Quick; Promega). The universal primer set 515f/806r targeting the V4 region of 16S rRNA genes was used. Both primers were modified to contain an Illumina adapter region and the reverse primer was encoded with 12-bp barcodes according to a previous report [46]. Thermal conditions of RT-PCR were the same as described previously [37], except that a total of 28 cycles were employed during PCR. The absence of DNA contamination was confirmed by the absence of amplification in the absence of reverse transcriptase. Illumina sequencing was conducted as shown in Supplementary Information (SI).

#### Sequence data processing

The PhiX, low-quality (*Q* < 30) and chimeric sequences were removed and the paired-end sequences were assembled as described previously [37, 47]. The sequences in each library were characterized phylogenetically using the QIIME software package version 1.7.0 [48]. An operational taxonomic unit (OTU) was defined using a cut-off of 97% sequence identity. Relative abundances in the OTUs were determined in both the ^13^C and the unlabeled treatments, and the statistical significance of their difference was calculated using the Student’s *t*-test. The OTUs that were more abundant in the heavy fractions of the ^13^C treatments than in the unlabeled treatment were phylogenetically identified with the BLAST program in the NCBI nucleotide sequence database (http://www.ncbi.nlm.nih.gov).

#### 16S rRNA expression assays of ^13^C-incorporating OTUs throughout SIP incubation

Total RNAs extracted at hours 0, 2, 4, 6 and 8 of the ^13^C treatment were used as templates for RT-qPCR and RT-PCR followed by Illumina sequencing. As for RT-qPCR, the total 16S rRNA copy numbers were determined using the universal primer set 515f/806r [46] with a GoTaq one-step RT-qPCR kit (Promega). Thermal conditions of RT-qPCR were the same as described previously [37]. Fluorescence was detected at the end of each extension step. In order to obtain a standard curve for quantification, 16S rRNA genes from *Escherichia coli* were amplified by PCR with the primer set B27f/B907r [49] and a serial dilution series (10^4^ to 10^8^ copies) of the plasmid-inserted PCR amplicon was prepared [50]. The procedures for RT-PCR, Illumina sequencing and data processing were as described above. The 16S rRNA molecules from the ^13^C-incorporating OTUs were estimated by the total bacterial 16S rRNA copy numbers (copies ml^−1^) and relative abundances (%) of the OTUs that were obtained by RT-qPCR and Illumina sequencing, respectively.

### Monitoring of the identified 1,4-dioxane degraders in a full-scale treatment system

#### Operation and physicochemical analyses of the full-scale system

The full-scale activated sludge system (Fig. S1) at NIPPON SHOKUBAI Co., Ltd. (Kawasaki, Japan) consisted mainly of a 450 m^3^ aeration tank and treated nearly 450 m^3^ day^−1^ of petrochemical industrial wastewaters containing high concentrations of 1,4-dioxane, mono-ethylene glycol and alkanes (e.g., undecane, dodecane, tridecane and tetradecane). The concentrations of 1,4-dioxane were 5.0−69.1 mg l^−1^, corresponding to 0.87–12.12% of the influent total organic carbon (TOC). The average hydraulic retention time was 1 day and the solids retention time for this system was 28 days. Dissolved oxygen (DO) in the aeration tank was monitored with a DO meter (DO5509; FUSO, Japan). Temperature and pH were determined with a thermometer (Yokogawa, Japan) and a pH meter (pH200S; Yokogawa), respectively. MLSS was determined with a MLSS meter (SS-5Z; Kasahara Chemical, Japan). TOC concentrations in the influent and effluent were measured by a TOC analyzer (TOC-4110; Shimadzu). The 1,4-dioxane concentration and COD of the activated sludge water were determined as described above. Long-term monitoring was performed for approximately 1 year, from May 2015 to April 2016, during which the system operation was stopped from 20th August to 23rd September for periodic maintenance. The activated sludge samples were taken from the aeration tank and return line, and stored at −20 °C for the microbial community analysis.

#### Fine-scale phylogenetic analyses of microbial communities in the full-scale system

According to the procedure mentioned above, total nucleic acids were extracted from 41 sludge samples in the aeration tank and 41 sludge samples in the return line. RNA was digested using an RNase (Type II-A; Sigma-Aldrich). The purified DNA was used as a template for PCR amplification with a Q5 High-Fidelity DNA polymerase (New England Biolabs, Japan). The primer set 515f/806r for Illumina sequencing was utilized, as mentioned. Thermal conditions of PCR were the same as described previously [41, 51], except that a total of 28−35 cycles were employed for amplification. Illumina sequencing was conducted as shown in SI. The sequence data processing was performed as described above, except for the alpha- and beta-diversity analysis whose procedure is described in SI.

### Nucleotide sequence accession numbers

All the sequences of 16S rRNA molecules and genes obtained from high-sensitivity SIP and Illumina sequencing of microbial communities in the full-scale treatment system have been deposited at the DNA Data Bank of Japan (DDBJ) under the accession numbers DRA005557 (206 libraries) and DRA005982 (15 libraries), and 16S rRNA genes of the 10 ^13^C-incorporating OTUs were deposited under accession numbers LC312382 to LC312391.

## Results

### Aerobic degradation of 1,4-dioxane by activated sludge

**Fig. 1:**
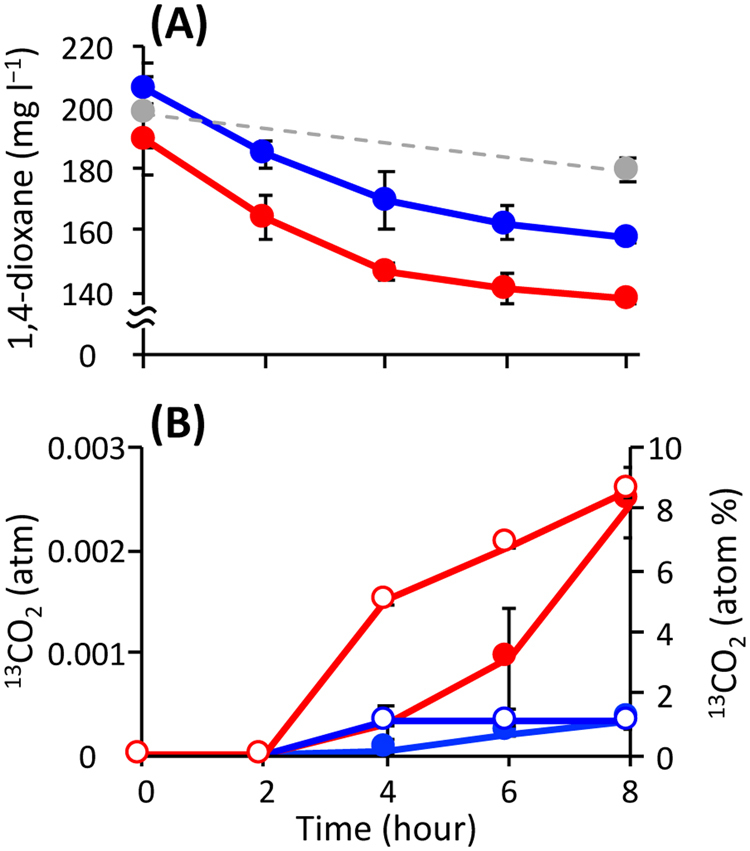
Changes in chemical parameters during aerobic incubation of activated sludge microorganisms with ^13^C-labeled and unlabeled 1,4-dioxane. Concentration of 1,4-dioxane **a**, ^13^C atom percentage of CO_2_ (**b**: open symbols) and calculated concentration of ^13^CO_2_ (**b**: closed symbols). The colors red, blue and gray indicate the ^13^C, unlabeled and heat-killed treatments, respectively. The error bars indicate the standard deviations of three replications

In the ^13^C and unlabeled treatments, concentrations of 1,4-dioxane in the liquid phases decreased by 49−51 mg l^−1^ during the incubations (Fig. 1a), and these decreases were accompanied with decreases of 77−101 mg l^−1^ in COD (Fig. S2A). No significant decrease in the 1,4-dioxane concentration was observed in the heat-killed treatment. Total CO_2_ in gaseous phases of the ^13^C and unlabeled treatments gradually increased after hour 2 to the concentrations of 0.028−0.031 atm at hour 8 (Fig. S2C). The fate of ^13^C-labeled 1,4-dioxane was traced by monitoring the ^13^C atom percentage of the gaseous product CO_2_ over time (Fig. 1b). The ^13^C atom percentage of CO_2_ increased drastically after hour 2, reaching a maximum value of 8.6% at hour 8 in the ^13^C treatment, whereas it remained constant at low levels (i.e., <1.1%) in the unlabeled treatment. The calculated ^13^CO_2_ exhibited a rapid increase after hour 2 in the ^13^C treatment (Fig. 1b). IC concentrations in the ^13^C treatment were almost constant (i.e., 136.0−142.4 mg l^−1^) (Fig. S2B). Concentrations of the headspace O_2_ ranged from 0.186 to 0.211 atm during the incubations (Fig. S2D), suggesting that a sufficient amount of O_2_ was provided for microbial degradation of 1,4-dioxane. These results indicated that the added ^13^C-labeled 1,4-dioxane was aerobically degraded to ^13^CO_2_ by the metabolic activities of the sludge microorganisms.

### Phylogenetic identification of the sludge microorganisms incorporating 1,4-dioxane-^13^C

**Fig. 2:**
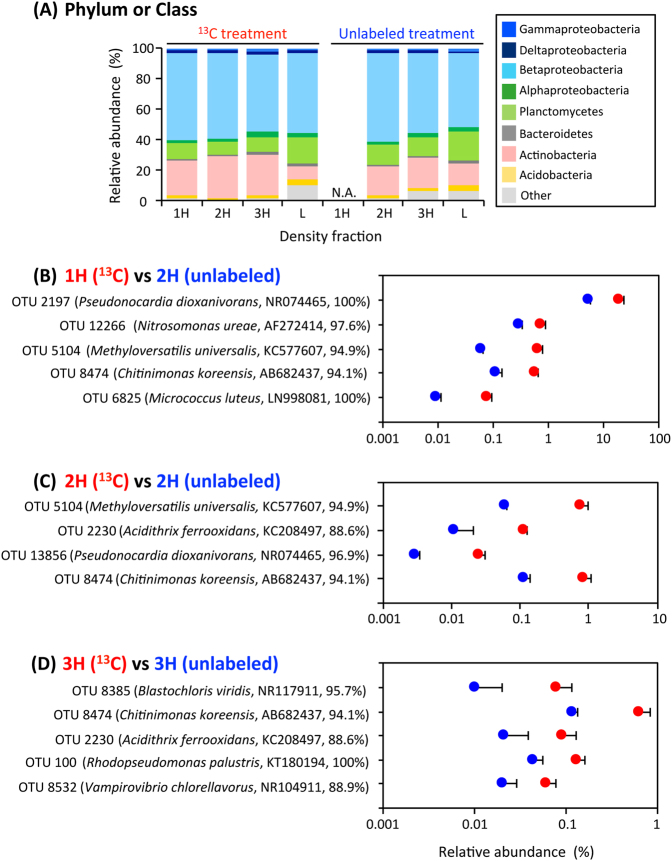
Identification of the ^13^C-incorporating microorganisms during the incubation with ^13^C-labeled 1,4-dioxane. **a** The phylum- and class-level distribution of density-resolved RNAs (*n* *=* 3). Phylogenetic groups are indicated by colors and their taxonomies are shown at the right side of the graph. The examined RNA density gradients are indicated as the 1H (heaviest), 2H (second-heaviest), 3H (third-heaviest) and L (light) fractions. The CsTFA BDs of the RNA density fractions and the total sequence numbers in the Illumina sequence libraries are summarized in Table S1. N.A. indicates no amplification product from RT-PCR. **b**–**d** The significantly ^13^C-incorporating OTUs. The OTUs that exhibited significantly high relative abundances in the 1H (**b)**, 2H (**c)** and 3H (**d**) fractions of the ^13^C treatment compared with those in the corresponding fractions of the unlabeled treatment (*n* = 3, *p* < 0.05) are shown. The closest relatives of the OTUs are indicated. The colors red and blue indicate the ^13^C and unlabeled treatments, respectively. The error bars indicate the standard deviations of three replications

We phylogenetically characterized the 1H, 2H, 3H and L fractions of RNA from the ^13^C treatment by high-throughput Illumina sequencing. Only the 2H, 3H and L fractions were subjected to Illumina sequencing in the unlabeled treatment, because the 1H fraction could not be amplified by RT-PCR, due to the low concentrations of the template RNA, indicating that there was little migration of unlabeled RNAs during ultra-centrifugation. The total number of 16S rRNA molecules obtained from 21 density fractions was around 1.34 million, corresponding to an average of 63,999 sequences per library (Table S1). A large quantity of Illumina sequence data is essential for the unambiguous detection of a small amount of ^13^C-labeled RNAs [37]. Phylum and class-level phylogenetic analysis indicated that the relative abundances of the phylum Actinobacteria of the heavy fractions were 2.7−3.3 times higher than those of the light fraction in the ^13^C treatment, but no other obvious changes accompanied the increase in BDs in either treatment (Fig. 2a). Nevertheless, our high-resolution OTU-level survey indicated that 10 microbial OTUs exhibited significantly higher relative abundances in the ^13^C treatment than in the unlabeled treatment (Fig. 2b–d). Specifically, in the 1H fraction (BD: 1.805 g ml^−1^) of the ^13^C treatment, the relative abundances of the OTUs 2197, 12266, 5104, 8474 and 6825 were 20.038, 0.747, 0.660, 0.566 and 0.080%, which were 3.7-, 2.5-, 11.0-, 4.9- and 8.7-fold higher than those in the 2H fraction of the unlabeled treatment (all significant at *P* < 0.03, *n* = 3; Fig. 2b). The OTUs 5104, 2230, 13856 and 8474 made up 0.782, 0.116, 0.025 and 0.834% of the total population in the 2H fraction (BD: 1.796 g ml^−1^) of the ^13^C treatment, and these percentages were 13.0-, 10.7-, 8.5- and 7.3-fold higher than those of the unlabeled treatment (all significant at *P* < 0.03, *n* = 3; Fig. 2c). The 3H fraction (BD: 1.790 g ml^−1^) of the ^13^C treatment showed 8.0-, 5.4-, 4.4-, 3.0- and 3.0-fold higher abundances of the OTUs 8385, 8474, 2230, 100 and 8532 (i.e., 0.081, 0.645, 0.093, 0.134 and 0.061%) than those of the unlabeled treatment (all significant at *P* < 0.04, *n* = 3; Fig. 2d). Among them, the OTUs 2230, 5104 and 8474 were identified more than once to be significantly enriched in ^13^C by the independent assays of the 1H, 2H and 3H fractions, affirming the validity of our criteria for the high-sensitivity SIP.

The 10 ^13^C-incorporating OTUs were phylogenetically diverse (Fig. 2b–d), or widely distributed in the phylogenetic trees (Fig. S3). In the case of the Actinobacteria species, the OTUs 2197 and 13856 were phylogenetically identical and similar, respectively, to the well-known 1,4-dioxane degrader *P. dioxanivorans* (accession no. NR074465; 100 and 96.9% sequence similarities). The OTU 2197 and *P. dioxanivorans* were located separately in the phylogenetic tree, likely due to the different lengths of the 16S rRNA sequences analyzed (Fig. S3A). This situation was also found for the phylogenic positions of the OTUs 6825 and 100 (Fig. S3A, S3C). The OTU 6825 was identical to *Micrococcus luteus* (LN998081; 100%). The OTU 2230 had a very low sequence similarity (88.6%) to the cultured relative *Acidithrix ferrooxidans* (KC208497) and fell into a specific clade with uncultured bacteria (e.g., DH092107_RO_07E [KC358648] and SSIM-F1v [FJ946539]) (Fig. S3B). Concerning the betaproteobacterial species, the OTU 12266 was the most related to *Nitrosomonas ureae* (AF272414; 97.6%) and was affiliated with the *Nitrosomonas* clade (Fig. S3D). The OTUs 5104 and 8474 were related to some extent to *Methyloversatilis universalis* (KC577607; 94.9%) and *Chitinimonas koreensis* (AB682437; 94.1%), and together formed a distinctive cluster together with uncultured bacteria (e.g., Anxy6 [HQ343211] and 5–25 [JQ923516]). The alphaproteobacterial OTU 8385 was related to *Blastochloris viridis* (NR117911; 95.7%) but fell into a specific clade not with *B. viridis* but rather Methylocystaceae bacterium PKR-39 (KJ000026) and uncultured bacteria (e.g., QEDN4CG12 [CU92524] and J22 [HQ697480]), whereas the OTU 100 was identical to *Rhodopseudomonas palustris* (KT180194; 100%) (Fig. S3C). The cyanobacterial OTU 8532 had a very low sequence similarity (88.9%) to the cultured relative *Vampirovibrio chlorellavorus* (NR104911), and in fact was quite distant from all cultured relatives in the phylogenetic tree (Fig. S3E).

**Fig. 3:**
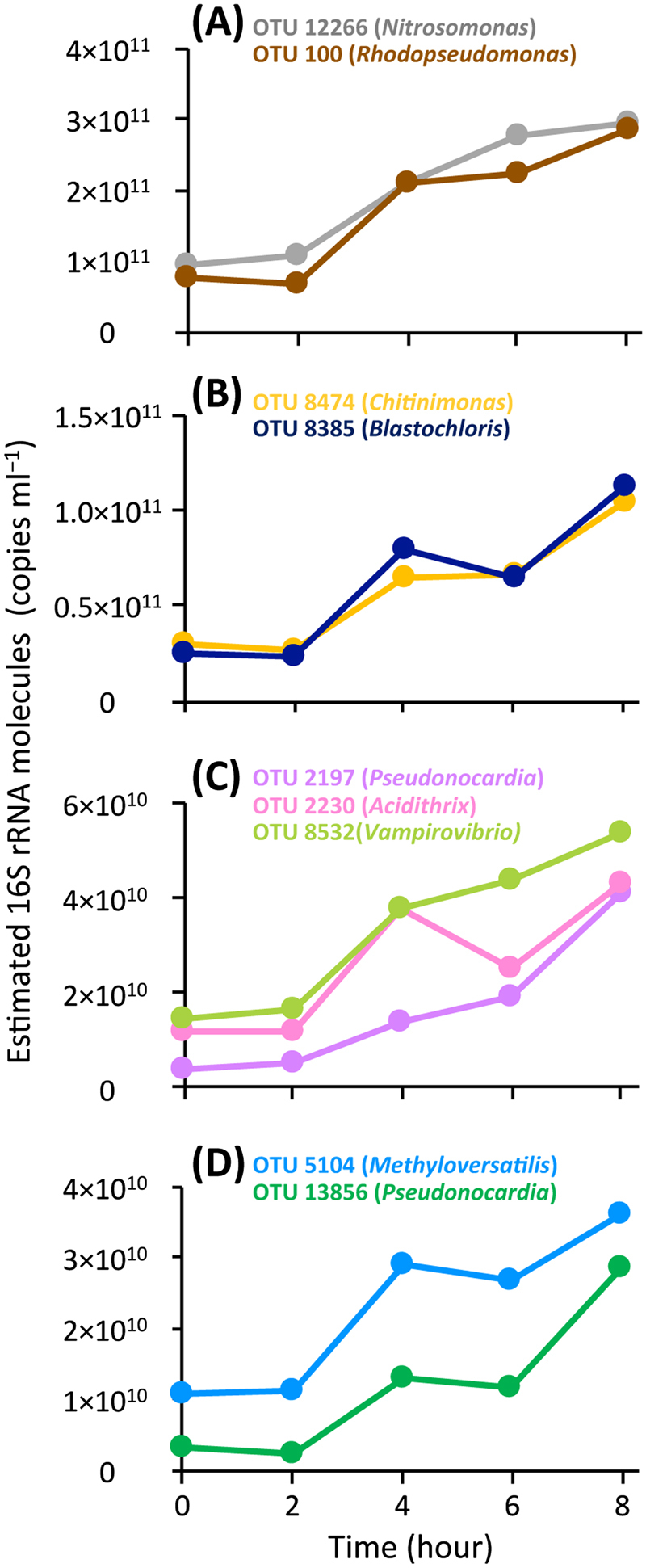
16S rRNA expression of the ^13^C-incorporating OTUs during the incubation with ^13^C-labeled 1,4-dioxane. **a**–**d** Estimated 16S rRNA molecules from the ^13^C-incorporating OTUs in the SIP experiment. Genera of the closest relatives of the OTUs are indicated in the parentheses of explanatory notes. 16S rRNA molecules from the OTUs were estimated by the total bacterial 16S rRNA copy numbers (copies ml^−1^) and relative abundances (%) of the OTUs that were determined by RT-qPCR (*n* = 3) and Illumina sequencing (*n* = 3), respectively. The details of the Illumina sequence libraries are summarized in Table S2

To address the metabolic activities of the 10 ^13^C-incorporating OTUs, the quantity of their 16S rRNA molecules was monitored throughout the ^13^C treatment (Fig. 3). The 16S rRNA molecules from the OTUs 12266, 2197 and 8532 linearly increased from hour 2, whereas those from six other OTUs (i.e., 5104, 8474, 2230, 13856, 8385 and 100) showed a two-step increase, that is, they increased temporally during hours 2−4 and 6−8. However, no expression of 16S rRNA from the OTU 6825 was found. Although potentially different in terms of metabolic induction, the nine OTUs became metabolically active promptly after the supplementation of 1,4-dioxane as the sole substrate, indicating these OTUs could directly degrade 1.4-dioxane. The range of their estimated 16S rRNA molecules was 2.42 × 10^9^−2.95 × 10^11^ copies ml^−1^, which was apparently not linked with their enrichment in ^13^C, that is, their relative abundances in the 1H, 2H and 3H fractions of the ^13^C treatment. This implied that the total metabolic activities and assimilative activities of 1,4-dioxane-^13^C differed from one species to another, possibly depending on the difference in the metabolic strategies employed. Consequently, a total of nine OTUs, including the 1,4-dioxane-degrading *P. dioxanivorans*, an ammonia-oxidizing bacterium and phylogenetically novel bacteria, were heavily enriched with 1,4-dioxane-derived ^13^C and further exhibited an increased expression of 16S rRNAs from the beginning of the SIP incubation, indicating their involvement in the degradation of ^13^C-labeled 1,4-dioxane.

### Dynamics of the physicochemical parameters and whole microbial communities in a full-scale treatment system

The efficiency of 1,4-dioxane removal fluctuated dramatically in parallel with changes in temperature and DO throughout the operation, although the TOC removal ratio and pH were rather stable (Fig. S5B−S5D). Illumina sequencing of 16S rRNA genes from 82 different activated sludge samples from the aeration tank and return line produced a total of 5.9 million sequences, corresponding to an average of 72,170 sequences per library (Table S3). Alpha diversity indices indicated that the microbial richness and evenness were almost the same between the tank and line, and these indices randomly shifted in certain ranges during the operation (Chao1: 1,779−11,131; Shannon: 2.8−7.0; Simpson reciprocal: 1.9−37.2). A principal coordinate analysis (PCoA) plot based on weighted UniFrac distances revealed that the whole microbial communities in the tank and line exhibited similar transition patterns (Fig. S6). Specifically, the microbial communities changed progressively throughout the operation, but those just after the system shutdown (upper right of the plot: dates 2nd, 13th and 19th October) were distinguishable from those on the other dates. In accordance with the irregular fluctuations in the α and β diversities, the phylum and class-level phylogenetic distribution show that the microbial communities changed drastically over the year of operation (Fig. S7). The dominant phylogenetic groups, that is, the phyla Acidobacteria, Actinobacteria, Armatimonadetes and Bacteroidetes and the classes Alpha- and Beta-proteobacteria, fluctuated but kept their relative abundances of >10%. These results implied that the roles of whole microbial communities in 1,4-dioxane removal could be limited, which in turn strongly suggested that the nine 1,4-dioxane-degrading OTUs identified earlier served as a functional guild in the activated sludge system.

### High-resolution dynamics of the 1,4-dioxane degraders in relation to the treatment system reactor performance

**Fig. 4:**
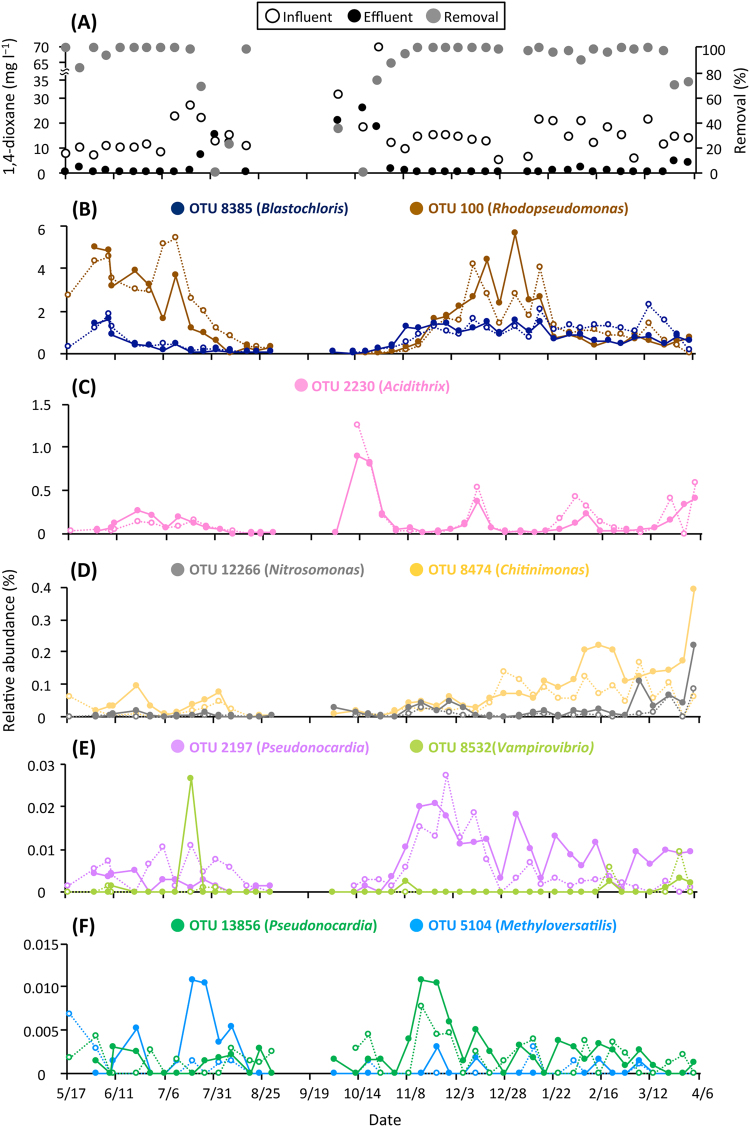
Changes in the 1,4-dioxane-related parameters and identified 1,4-dioxane degraders in the full-scale activated sludge system. **a** 1,4-dioxane-related parameters, that is, concentrations of 1,4-dioxane (open symbols, influent; closed symbols, effluent) and 1,4-dioxane removal efficiency (gray-color symbols). **b**–**f** Relative abundances of the identified 1,4-dioxane degraders in the aeration tank (closed symbols and solid lines) and return line (open symbols and dotted lines) of the full-scale treatment system. Genera of the closest relatives of the OTUs are indicated in the parentheses of explanatory notes. The abundance of each OTU was determined by Illumina sequencing of 16S rRNA genes. The details of the Illumina sequence libraries are summarized in Table S3

With the high-sensitivity SIP results providing the phylogenetic identities of 10 OTUs incorporating 1,4-dioxane-^13^C, we extracted the data on the relative abundances of these OTUs from the Illumina sequence libraries. The OTU 6825 was not detected throughout the operation, indicating that this OTU was not a member of functional guild of 1,4-dioxane degraders, which is consistent with the results from 16S rRNA expression analysis of the SIP incubation. The other nine OTUs, 2197, 12266, 5104, 8474, 2230, 13856, 8385, 100 and 8532, were detected in the aeration tank at the yearly averaged relative abundances of 0.007, 0.021, 0.001, 0.074, 0.137, 0.002, 0.682, 1.523 and 0.001%, respectively. In addition, the individual OTUs showed similar successions in the aeration tank and return line (Fig. 4b–f). Changes in the absolute abundances of the nine OTUs were determined based on MLSS, that is, total biomass, of the tank and line (Fig. S9B−S9F), which showed a quite similar trend to the relative abundance changes (Fig. 4b–f). Although 1,4-dioxane degradation efficiency did not represent the actual removal capability because of the non-constant concentrations of 1,4-dioxane in the influent, the dynamics of the nine identified OTUs in the tank were tightly associated with changes in the 1,4-dioxane removal efficiency (Fig. 4, S9 and Table S4). In particular, the removal efficiencies of 1,4-dioxane were high (98.7–99.2%) when the temperature was held nearly constant at <30.7 °C, and the DO levels were 2.7–6.4 mg l^−1^ from 17th May to 6th July (except on 25th May [83.7%] and 8th June [93.6%]), when relatively high abundances of the OTUs 8385 and 100 (0.184–5.120%) were noted. The decreases in performance on 25th May and 8th June may have been related to a temporal decrease in the influent TOC to 47.4 mg l^−1^ and the resultant high ratio (8.3%) of 1,4-dioxane to the TOC on 18th May . The OTUs 8385 and 100 were temporarily increased in response to the former decrease in efficiency and the OTUs 2230 and 8474 were temporarily increased in response to the latter decrease, which would have contributed to the rapid recovery of the removal efficiencies. From 6th to 21st July, a gradual increase in temperature to 33.0 °C and a sudden drop of DO to 1.1 mg l^−1^ coincided with lasting decreases in the OTUs 100, 8385 (Fig. S8A) and 2230 (Fig. S8C), but transient increases in the OTUs 8474, 8532 and 5104, thereby maintaining the high 1,4-dioxane removal efficiency (98.4%) on 21st July. However, from 27th July to 10th August, the inflows of 1,4-dioxane at 12.5−21.9 mg l^−1^ and TOC at 446.9–684.5 mg l^−1^, as well as the continued high temperatures (34.0 °C–35.4 °C) and low DO levels (0.9–1.7 mg l^−1^), resulted in low 1,4-dioxane removal efficiencies (0–68.2%). In this regard, the relative abundances of all the 1,4-dioxane degrader OTUs decreased to substantially low levels (0.001–0.079%) on 10th August. Thereafter, the decrease in temperature to 30.0 °C, increase in DO to 5.5 mg l^−1^ and lowered influent TOC of 183.4 mg l^−1^ on 19th August again led to a high 1,4-dioxane removal efficiency (98.0%), and under these conditions the OTUs 100 and 2230 appeared to increase (Fig. S8B and S8D). For about 10 days after the system restarted on 24th September, the temperature, DO and TOC removal ratio were stabilized to values similar to those before the system shutdown. However, despite the high DO levels (4.2–6.4 mg l^−1^) provided, the removal efficiencies of 1,4-dioxane were quite low (0–34.5%) from 5th to 19th October, during which the relative abundance of the OTU 2230 rapidly increased from 0.005% to 0.907%. In the days immediately after 19th October, a dramatic recovery from the performance decline was realized simultaneously with sharp increases in the OTUs 8385, 100 (Fig. S8A and S8B), 2197 and 13856. Due to the nearly constant temperatures (21.3 °C–30.2 °C) and sustained DO levels (3.7–5.9 mg l^−1^), the stable reactor performance with a high 1,4-dioxane removal efficiency of 95.4–99.4% continued from 16th November to 2nd February, in association with a variety of transition behaviors of the identified 1,4-dioxane degraders, that is, gradual increases for the OTUs 100 and 8474, maintenance at a constant level of the OTU 8385, fluctuations for the OTUs 2197 and 13856 and transient appearances for the OTUs 2230 and 12266. Moreover, a moderate decrease in 1,4-dioxane removal efficiency (89.2%) on 8th February was quickly recovered, concurrent with transient increases in the OTUs 2230, 8474 and 12266. These three OTUs increased gradually during the successive stable operation from 15th February to 22nd March, then more rapidly in response to the lowered 1,4-dioxane removal efficiencies (69.5–71.6%) during the end of the operation from 28th March to 4th April. These results indicate that the nine 1,4-dioxane-degrading OTUs co-existed in the tank at yearly averaged relative abundances of 0.001–1.523%, but showed dynamic and individually distinct transitions in response to changes in the 1,4-dioxane removal efficiency under changing conditions in the activated sludge system.

## Discussion

1,4-Dioxane is one of the most recalcitrant artificial pollutants in petrochemical industrial wastewaters and chlorinated solvent groundwater plumes [2]. As the first application of high-sensitivity rRNA-SIP to environmental samples, we herein investigated the identity and diversity of the activated sludge microorganisms involved in the degradation of 1,4-dioxane. Using GC-C-IRMS in parallel with chemical analyses, we clarified the fate of ^13^C-labeled 1,4-dioxane during SIP incubation across time. The environmentally relevant concentration of ^13^C-labeled 1,4-dioxane and short incubation time allowed the sludge microorganisms to incorporate the substrate-borne ^13^C under quasi-natural conditions. Although it is possible that some of the ^13^C-incorporating microorganisms could not degrade ^13^C-labeled 1,4-dioxane but rather utilized its degradative intermediates supplied by the other degraders, the increased 16S rRNA expression shortly after the addition of ^13^C-labeled dioxane as the sole substrate confirmed the key microorganisms that could directly metabolize 1,4-dioxane. Together, we identified a diverse range of active 1,4-dioxane degraders, although some degraders might be overlooked due to the controlled environmental variables in the SIP experiment. Furthermore, high-resolution phylogenetic monitoring over 1-year of operation of a full-scale activated sludge system highlighted an apparent link between the dynamics of the identified degraders and 1,4-dioxane removal efficiency.

During the SIP incubation, the concentrations of degraded 1,4-dioxane-^13^C and gaseous product ^13^C (i.e., ^13^CO_2_) were 0.0466 and 0.0144 mmol per vial, respectively (Fig. 1). Given that IC is present mostly in the form of bicarbonate in pH 8.2, the produced dissolved inorganic ^13^C was calculated as 0.0201 mmol per vial (Fig. S2B). The carbon recovery in the ^13^C-labeled 1,4-dioxane treatment was 74.04%, which was consistent with the ^14^C-labeled 1,4-dioxane turnover data [52], in which more than half of the 1,4-dioxane was mineralized to CO_2_ by cultured 1,4-dioxane degraders. The carbon not recovered was likely attributable to either the degradative intermediates of 1,4-dioxane remaining in the incubated sludge and/or the portion assimilated to the microbial biomass including rRNA, which became labeled with ^13^C. In spite of the relatively low concentration (i.e., 0.58 mmol l^−1^) of the ^13^C-labeled 1,4-dioxane converted, ^13^C-labeled RNAs, that is, the 1H, 2H and 3H fractions, were retrieved by ultra-centrifugation and fractionation (Table S1). The BDs 1.796−1.805 g ml^−1^ of the 1H and 2H fractions correspond to those of the RNA density fractions in which even low amounts of ^13^C-labeled rRNAs are significantly accumulated [37]. The BD 1.790 g ml^−1^ of the 3H fraction corresponds to the BD showing a moderate accumulation of ^13^C-labeled rRNAs [37]. High-throughput sequencing of these heavy fractions indicated that the 10 OTUs significantly incorporated the ^13^C-labeled 1,4-dioxane, and thus were enriched in ^13^C (Fig. 2b–d). Among them, the OTU 6825 was ruled out from the functional guild as a result of 16S rRNA expression assays. Based on the principle of density separation, the ^13^C enrichment of 16S rRNA molecules should be higher in the 1H and 2H fractions than in the 3H fraction. We speculate that the OTUs 2197, 12266, 5104 and 13856, which were identified exclusively in the 1H and 2H fractions, may have metabolized only ^13^C-labeled 1,4-dioxane, whereas the OTUs 8385, 100 and 8532, which were identified solely in the 3H fraction, may have co-metabolized ^13^C-labeled 1,4-dioxane in the presence of the other unlabeled compounds (e.g., mono-ethylene glycol and alkanes) that were originally contained in the activated sludge. Such a co-metabolism mechanism is supported by the previous finding that several short-chain alkanes were the primary carbon sources supporting the growth of the 1,4-dioxane degrader *Mycobacterium austroafricanum* strain JOB5 during co-metabolism [53]. By selecting these two distinct metabolic modes, the other OTUs 8474 and 2230 might flexibly adapt to the changing environmental conditions, for example, dynamic fluctuation of the ratio of 1,4-dioxane to the influent TOC in a wide range of 0.87–12.12% (Fig. 4a and S5D). In addition, the assimilation of ^13^C-labeled 1,4-dioxane into 16S rRNA molecules of the moderately ^13^C-enriched OTUs 8385, 100, 8532, 8474 and 2230 should be noted. There is a possibility that, through the co-metabolism of these OTUs, 1,4-dioxane-derived ^13^C was utilized not for growth but for maintenance (i.e., for the generation of 16S rRNA molecules) [54]. On the other hand, during the operation of the full-scale activated sludge system, the putative 1,4-dioxane co-metabolizer OTUs 8385, 100, 8532, 8474 and 2230 showed high yearly averaged relative abundances (the mean: 0.483%, the range: 0.001–1.523%), whereas the abundances of the putative metabolizer OTUs 2197, 12266, 5104 and 13856 were rather low (the mean: 0.008%, the range: 0.001–0.021%) (Fig. 3). It is tempting to imagine that the presumed metabolic modes of the identified 1,4-dioxane degraders affected their population size and dynamics in the system. However, the extent of ^13^C enrichment of 16S rRNA molecules is not yet confirmed as an index of the versatility of carbon source utilization of the degraders. Future investigations will be needed to clarify the ecophysiological futures and metabolic strategies of the identified 1,4-dioxane-degrading microorganisms.

It is noteworthy that the 1,4-dioxane removal efficiency of the full-scale treatment system was intrinsically linked with the dynamics of the identified 1,4-dioxane degraders (Fig. 4) but not with the dynamics of whole microbial communities (i.e., α-diversity indices, the PCoA plot based on weighted UniFrac distances, or phylum- and class-level phylogenetic data) (Figs. S6, S7 and Table S3). The heavily ^13^C-enriched OTUs 2197 and 13856 were phylogenetically identical and similar, respectively, to the well-known 1,4-dioxane degrader *P. dioxanivorans* (NR074465) (Fig. 2b, c and S3A). *P. dioxanivorans* can aerobically degrade 1,4-dioxane to CO_2_ as a sole carbon and energy source [55] and its degradation pathway has been proposed [56]. The transformation ability of *P. dioxanivorans* has been observed after the induction by 1,4-dioxane and its related compounds [25, 56, 57]. During the operation of the full-scale system, these OTUs showed similar annual transitions, especially with respect to their proliferation and continued existence under relatively stable aerobic conditions after 19th October (Fig. 4e, f and S5B). The detection of the confirmed 1,4-dioxane-degrading bacterium solidified the reliability of the high-sensitivity SIP results in this study. Aside from *P. dioxanivorans*, the OTU 100 was phylogenetically identical to *R. palustris* (KT180194), which has been shown to exhibit benzoate degradation ability and to operate a variety of oxygen-dependent enzymes [58]. Notably, this OTU was located in a phylogenetic cluster containing the 1,4-dioxane-degrading *Afipia* sp. strain D1 (accession no. AB586143) (Fig. S3C). The sequence similarity of OTU 100 and strain D1 was relatively high (96.4%). Whereas the other alphaproteobacterial OTU 8385 showed 95.7% similarity to *B. viridis* (NR117911), which grows unimpaired in the presence of dioxane [59], it was more closely related to and formed the same phylogenetic clade with Methylocystaceae bacterium PKR-39 (KJ000026; 96.5% similarity) (Fig. S3C), a relative of the obligate methanotroph *Methylosinus trichosporium* involved in the co-metabolism of 1,4-dioxane [24]. These two OTUs identified in the 3H fraction (Fig. 2d) were the dominant degraders in the actual system run, showing yearly averaged relative abundances of 1.523% (for the OTU 100) and 0.682% (for the OTU 8385) (Fig. 3b). Their proliferation was associated with high removal efficiencies of 1,4-dioxane. In this context, these aerobic organisms decreased remarkably under low DO conditions from 6th July to 10th August, triggering the decline in performance of the system. Further, the OTU 12266 belonged to *Nitrosomonas ureae* (AF272414; 97.6%), a chemolithotrophic ammonia-oxidizing bacterium [60]. It is vital to point out that the ammonia monooxygenase of the OTU 12266 would play a role in the first step of 1,4-dioxane degradation, because some of the cultured bacteria that express various types of monooxygenases are also involved in the metabolism and co-metabolism of 1,4-dioxane [24–26]. The OTUs 8474 and 5104 formed a distinctive phylogenetic cluster comprised of only uncultured bacteria, such as Wu-C65 (KJ783141) and Anxy6 (HQ343211), found in activated sludges (Fig. S3D), indicating that the constituent members of this cluster were likely candidates for hitherto unknown 1,4-dioxane degraders. The OTUs 2230 and 8532 have quite low similarities (<88.9%) to any cultured organisms, which makes it impossible to estimate their physiological functions. These five OTUs increased rapidly in response to transient decreases in 1,4-dioxane removal efficiencies, especially during the system restart after 24th September (for the OTU 2230) and the end of the operation from 28th March to 4th April (for the OTUs 8474 and 12266), thereby facilitating a recovery from the reduction in performance.

## Conclusion

Due to the high phylogenetic diversity of 1,4-dioxane degraders, which renders the tracing of the functional guild rather difficult, microbial transformation and degradation of 1,4-dioxane in natural environments are largely unknown while 1,4-dioxane degradation has been intensively studied by characterizing isolates and tracing biomarker genes [12]. In this study, high-sensitivity SIP was implemented to identify hitherto unknown but functionally important 1,4-dioxane degraders in natural environments. We discovered the 10 significantly ^13^C-incorporating microbial species from the sludge microorganisms by rigorously analyzing 16S rRNA molecules in RNA density fractions of the ^13^C-labeled and unlabeled 1,4-dioxane treatments. By employing a 16S rRNA expression assay, we showed that 9 of the 10 species, including the 1,4-dioxane-degrading *P. dioxanivorans* and an ammonia-oxidizing bacterium, increased their metabolic activities promptly after the onset of the SIP incubation, strengthening the argument that these ^13^C-incorporating microorganisms could directly degrade ^13^C-labeled 1,4-dioxane. The subsequent high-resolution phylogenetic analysis over the 1-year operation of a full-scale treatment system corroborated the presence and dynamics of these nine species as the functional guild that were tightly associated with the 1,4-dioxane degradation efficiency. The strategy we have taken is very effective for identification and ecophysiological characterization of the elusive 1,4-dioxane degraders. Based on the extent of their ^13^C enrichment in the SIP incubation and their relative abundances in the actual system run, we showed that each of these 1,4-dioxane degraders may possess a distinct metabolic mode, that is, metabolism or co-metabolism, which would not otherwise be accessible, under changing environmental conditions. Consequently, the co-existence and individually distinct dynamics of various 1,4-dioxane degraders, including hitherto unidentified species, played pivotal roles in the maintenance of this biological system for removing 1,4-dioxane. The global distribution and ecophysiological roles of these 1,4-dioxane degraders should be investigated to clarify the microbiological mechanism underlying the natural attenuation of the persistent pollutant.

## Electronic supplementary material

Supplementary information
