# Supplementary material for: Identification of active and taxonomically diverse 1,4-dioxane degraders in a full-scale activated sludge system by high-sensitivity stable isotope probing
Source: ISME J. 2018 Jun 13;12(10):2376–88. doi: 10.1038/s41396-018-0201-2 (PMC6155002; doi:10.1038/s41396-018-0201-2)
Supplement: Supplementary file 1 — Supplementary information [file 41396_2018_201_MOESM1_ESM.pdf]

Supplementary Information

Identification of active and taxonomically diverse 1,4-dioxane degraders in a full-scale activated sludge system by high-sensitivity stable isotope probing

Tomo Aoyagi[1†], Fumiaki Morishita[2†], Yutaka Sugiyama[2], Daisuke Ichikawa[2], Daisuke Mayumi[3], Yoshitomo Kikuchi[4], Atsushi Ogata[1], Kenji Muraoka[2], Hiroshi Habe[1] and Tomoyuki Hori[1†*]

[1] *Environmental Management Research Institute, National Institute of Advanced Industrial Science and Technology (AIST), 16-1 Onogawa, Tsukuba 305-8569, Japan*

[2] *NIPPON SHOKUBAI Co., Ltd., 14-1 Chidorichou, Kawasaki 210-0865, Japan*

[3] *Institute for Geo-Resources and Environment, Geological Survey of Japan, National Institute of Advanced Industrial Science and Technology (AIST) 1-1-1 Higashi, Tsukuba 305-8567, Japan*

[4] *Bioproduction Research Institute, National Institute of Advanced Industrial Science and Technology (AIST), 2-17-2-1 Tsukisamu-higashi, Sapporo 062-8517, Japan*

[†] T. Aoyagi, F. Morishita and T. Hori contributed equally to this work.

***Correspondence:** Tomoyuki Hori

Phone: +81 29 849 1107; Fax: +81 29 861 8326; e-mail: hori-tomo@aist.go.jp

Running title: Identification of 1,4-dioxane degraders by SIP

Keywords: high-sensitivity stable isotope probing; 1,4-dioxane; activated sludge; high-throughput sequencing; 16S rRNA

Supplementary Martials and Methods

Illumina sequencing

RT-PCR and PCR amplicons were purified first with an AMPure XP Kit (Beckman Coulter, CA, USA) and then with a Wizard SV gel and PCR clean-up kit (Promega, Japan). DNA concentrations of the purified amplicons were determined spectrophotometrically with a Quant-iT PicoGreen dsDNA reagent and kit (Invitrogen, CA, USA) and a NanoDrop3300 fluorospectrometer (Thermo Fisher Scientific, MA, USA). An appropriate amount of the purified amplicons (i.e., the barcode-encoded libraries) and an internal control (PhiX Control V3; Illumina, CA, USA) was subjected to paired-end sequencing with a 300-cycles MiSeq Reagent kit (Illumina) and a MiSeq sequencer (Illumina).

Construction of phylogenetic trees

In addition, phylogenetic trees showing the relationships of the significantly ^{13}C-incorporating OTUs were constructed using an ARB software package (http://www.arb-home.de) (Ludwig *et al*., 2004) as described previously (Hori *et al* ., 2010). Phylogenetic core trees were constructed from reference 16S rRNA gene sequences (> 1,200 nucleotides) using neighbor-joining, maximum-parsimony and maximum-likelihood algorithms. These different algorithms did not have a significant effect on the tree topology. Partial 16S rRNA sequences (approximately 250 bp) of the ^{13}C-enriched OTUs were aligned against the Silva v123 databases using SINA 1.2.11 (Pruesse *et al*., 2012) and added to the core trees using the ARB-parsimony tool.

Alpha- and beta-diversity analysis

Alpha diversity indices (i.e., Chao1, Shannon and Simpson reciprocal) and the weighted UniFrac distances for principal coordinate analysis were calculated on the basis of an equal number (n= 28,802) of sequences using the software package QIIME (Caporaso *et al*., 2010). The Chao1 index is used to represent the richness of microbial communities for prediction of

the total number of phylogenetically distinct species (Chao, 1984), and the indices Shannon and Simpson reciprocal mainly reflect the evenness and richness of microbial communities, emphasizing the effects of minor and predominant species, respectively (Shannon, 1948; Simpson, 1949).

References

- Caporaso JG, Kuczynski J, Stombaugh J, Bittinger K, Bushman FD, Costello EK *et al* (2010). QIIME allows analysis of high-throughput community sequencing data. *Nat Methods* **7:** 335-336.
- Chao A (1984). Nonparametric Estimation of the Number of Classes in a Population. *Scand J Stat* **11:** 265-270.
- Hori T, Muller A, Igarashi Y, Conrad R, Friedrich MW (2010). Identification of iron-reducing microorganisms in anoxic rice paddy soil by ^{13}C-acetate probing. *The ISME J* **4:** 267-278
- Ludwig W, Strunk O, Westram R, Richter L, Meier H, Yadhukumar et al (2004). ARB: a software environment for sequence data. *Nucleic Acids Res* **32:** 1363-1371.
- Pruesse E, Peplies J, Glöckner FO (2012). SINA: Accurate high-throughput multiple sequence alignment of ribosomal RNA genes. *Bioinformatics* **28:** 1823-1829.
- Shannon CE (1948). A Mathematical Theory of Communication. *Bell Syst Tech J* **27:** 623-656.
- Simpson EH (1949). Measurement of diversity. *Nature* **163:** 688-688.

Supplementary Figure S1: Schematic overview of the full-scale activated sludge system

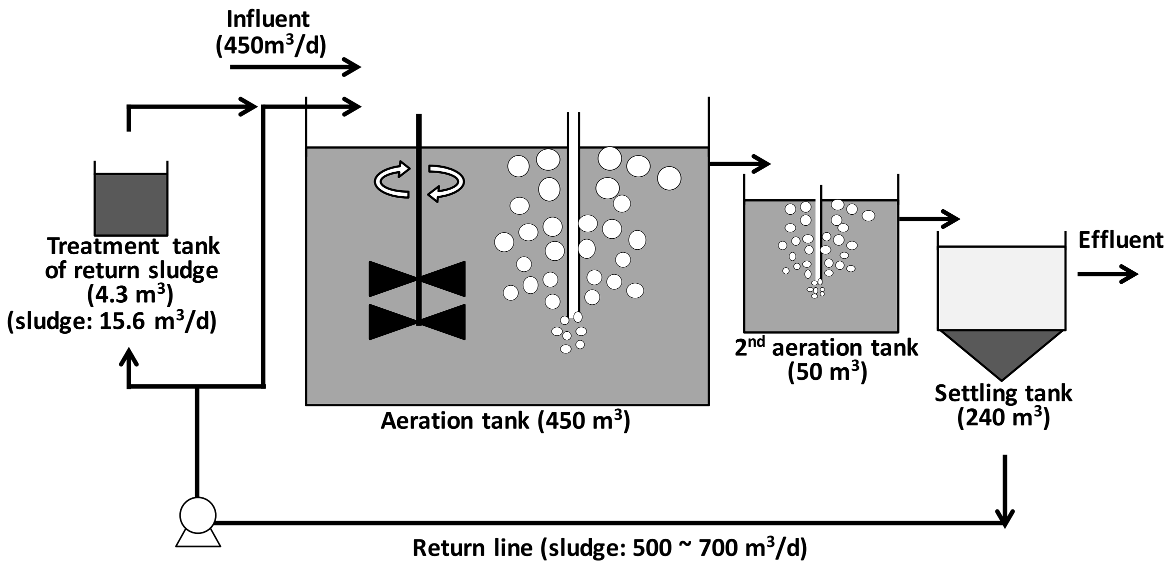

Supplementary Figure S2: Changes in chemical parameters during aerobic incubation of activated sludge microorganisms with the ^{13}C-labeled and unlabeled 1,4-dioxane

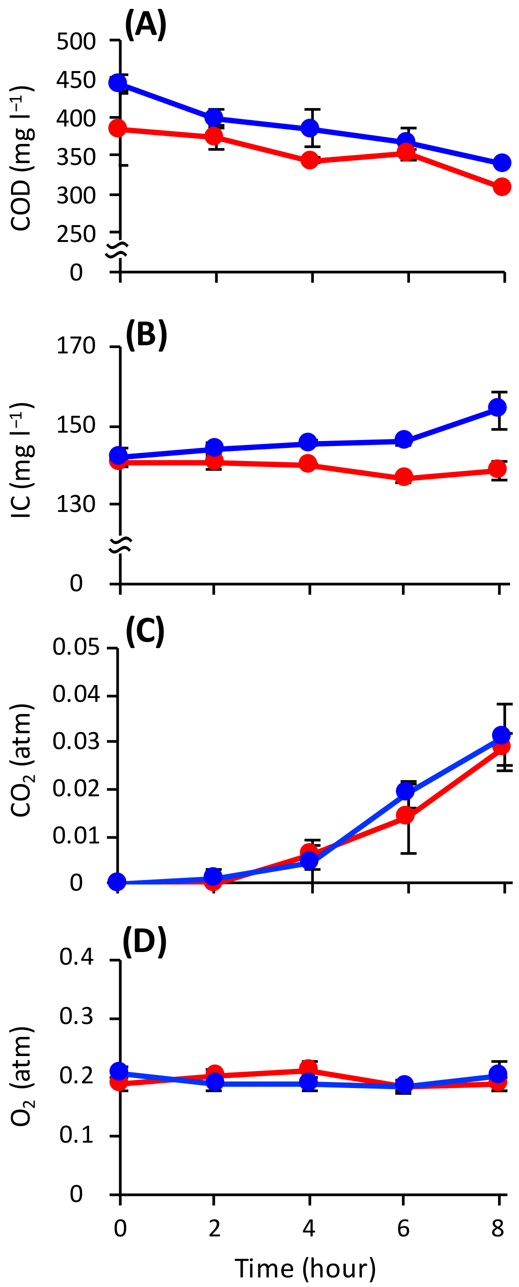

(**A**) Concentration of chemical oxygen demand (COD), (**B**) concentration of inorganic carbon (IC), (**C**) concentration of total CO_2, and (**D**) concentration of O_2. The colors red and blue indicate the ^{13}C and unlabeled treatments, respectively. The error bars indicate the standard deviations of three replications.

Supplementary Figure S3: Phylogenetic trees showing the relationships of the significantly ^{13}C-incorporating OTUs during the incubation with ^{13}C-labeled 1,4-dioxane

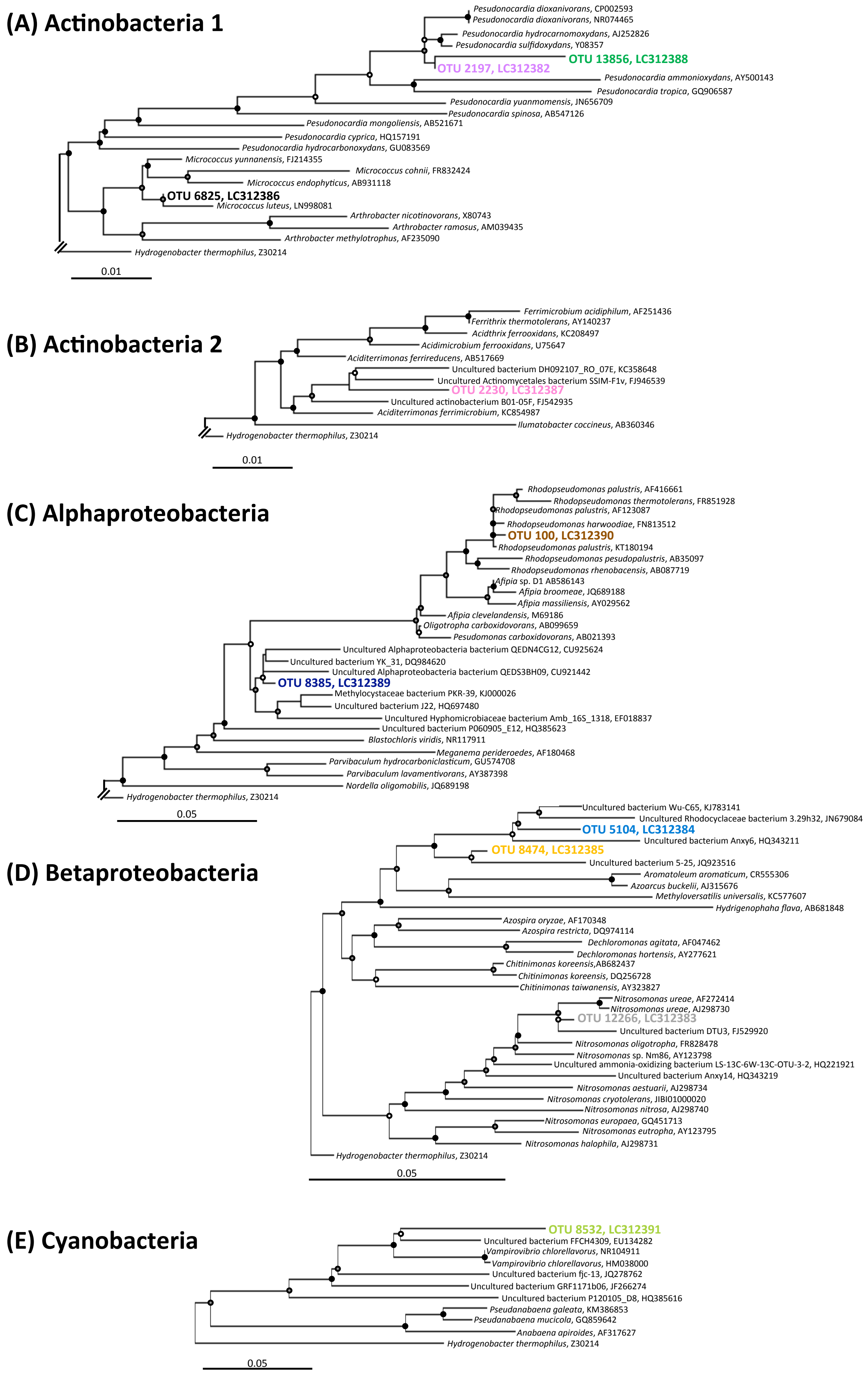

Phylogenetic trees showing the relationships of the OTUs affiliated with the Actinobacteria (**A, B**), Alphaproteobacteria (**C**), Betaproteobacteria (**D**) and Cyanobacteria (**E**). The core tree of reference sequences was reconstructed using the neighbor joining method based on a comparison of more than 1,200 nucleotides. Bootstrap values were obtained from 1,000 replications, and >90%, 75% to 89%, and 50% to 74% are shown with black, gray, and open circles, respectively. Sequences obtained in this study are indicated in colors and boldface. The scale bars represent 1% and 5% sequence divergences. GenBank accession numbers of sequences are given.

Supplementary Figure S4: 16S rRNA expression profiles of the ^{13}C-incorporating OTUs and microbial communities during the incubation with ^{13}C-labeled 1,4-dioxane

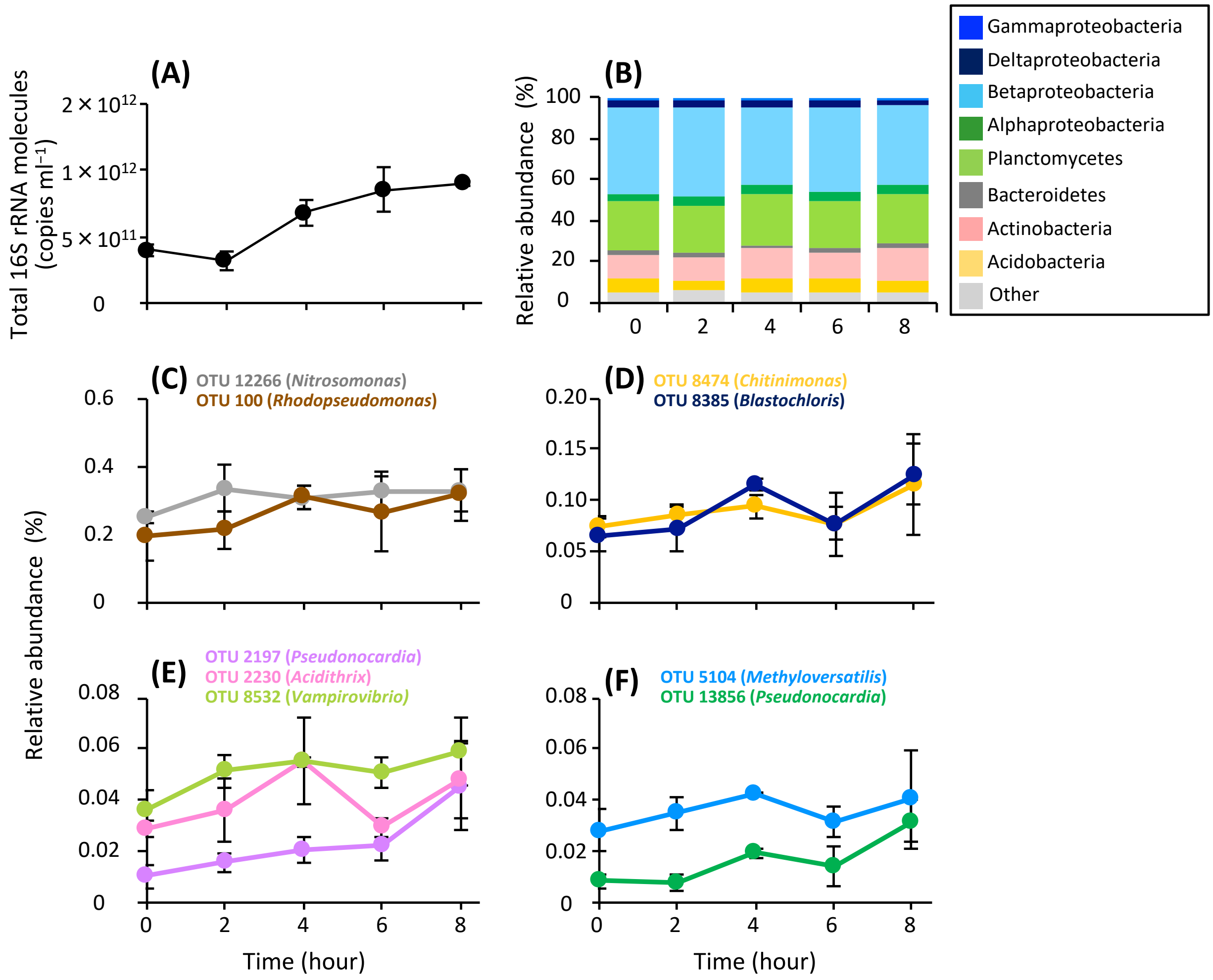

(**A**) Total 16S rRNA copy numbers as determined by RT-qPCR analysis. (**B**) The phylum and class level distribution of 16S rRNA molecules from whole microbial communities. Phylogenetic groups are indicated by colors and their taxonomies are shown at the right side of the graph. (**C**−**F**) Relative abundances of the ^{13}C-incorporating OTUs. Genera of the closest relatives of the OTUs are indicated in the parentheses of explanatory notes. The abundances of each OTU were determined by Illumina sequencing of 16S rRNA molecules. The details of the Illumina sequence libraries are summarized in Table S2. The error bars indicate the standard deviations of three replications.

Supplementary Figure S5: **Variations in reactor performances during the operation of the full-scale activated sludge system**

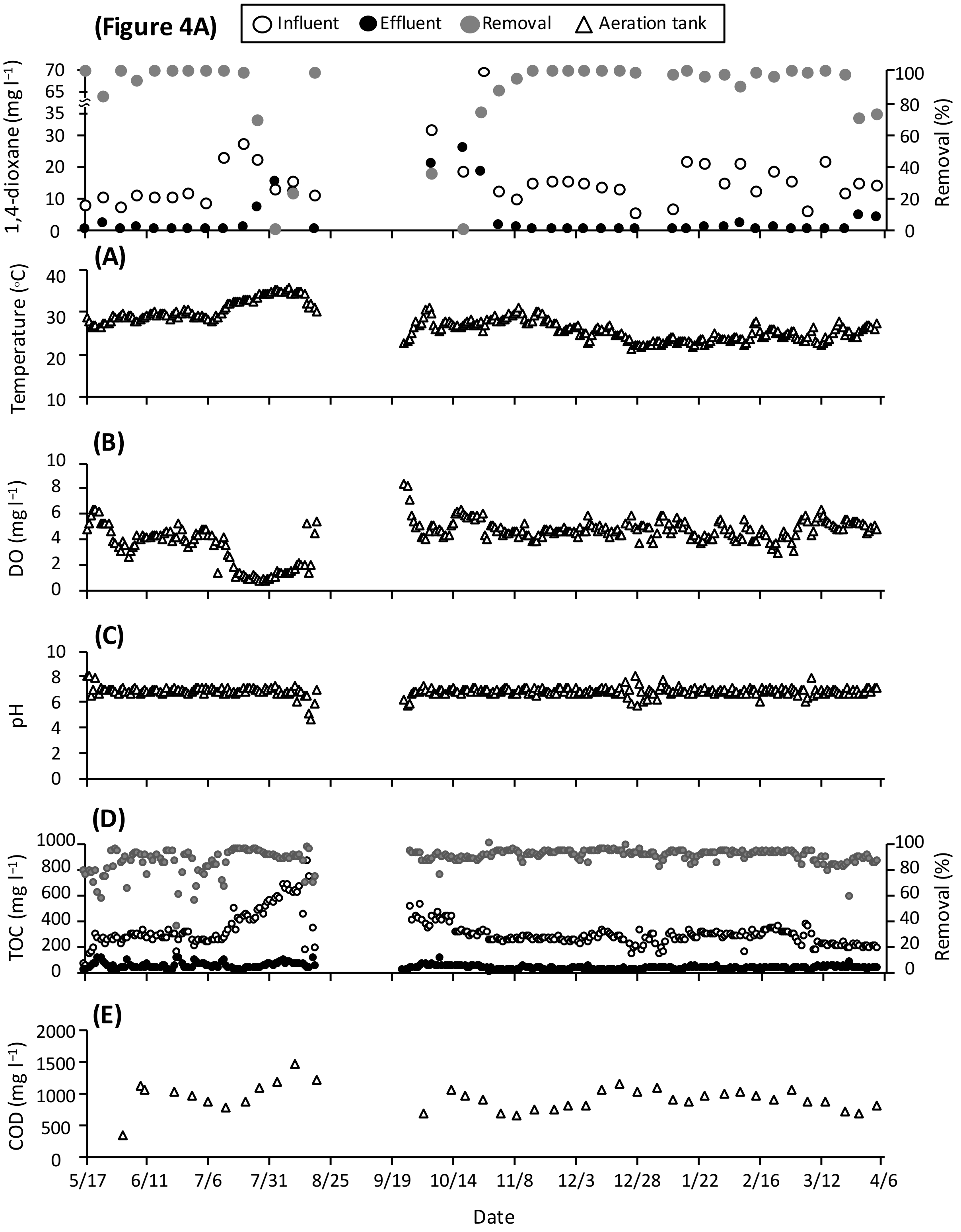

Time course of physicochemical parameters in the full-scale treatment system. (**A**) Temperature, (**B**) concentration of dissolved oxygen (DO), (**C**) pH, (**D**) concentrations of total organic carbon (TOC) (open symbols, influent; closed symbols, effluent) and TOC removal ratio (gray-color symbols), (**E**) concentration of chemical oxygen demand (COD).

Supplementary Figure S6: **Principal coordinate analysis (PCoA) plot showing the microbial community succession during the operation of the full-scale activated sludge system**

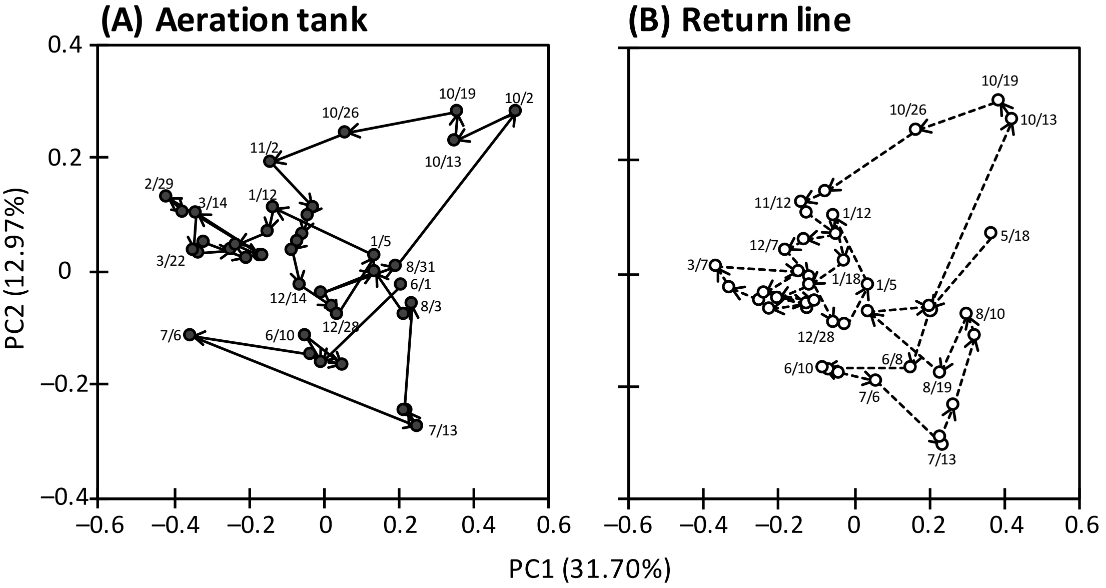

PCoA plot of the Illumina sequence data of 16S rRNA genes from the aeration tank (**A**) and return line (**B**) of the full-scale treatment system. The PCoA plot was generated from the weighted UniFrac analysis based on an equal number (*n*= 28,802) of sequences. The sampling dates are indicated. The arrows indicate the temporal progress. The details of the Illumina sequence libraries are shown in Table S3.

Supplementary Figure S7: Phylum and class level distribution of microbial communities during the operation of the full-scale activated sludge system

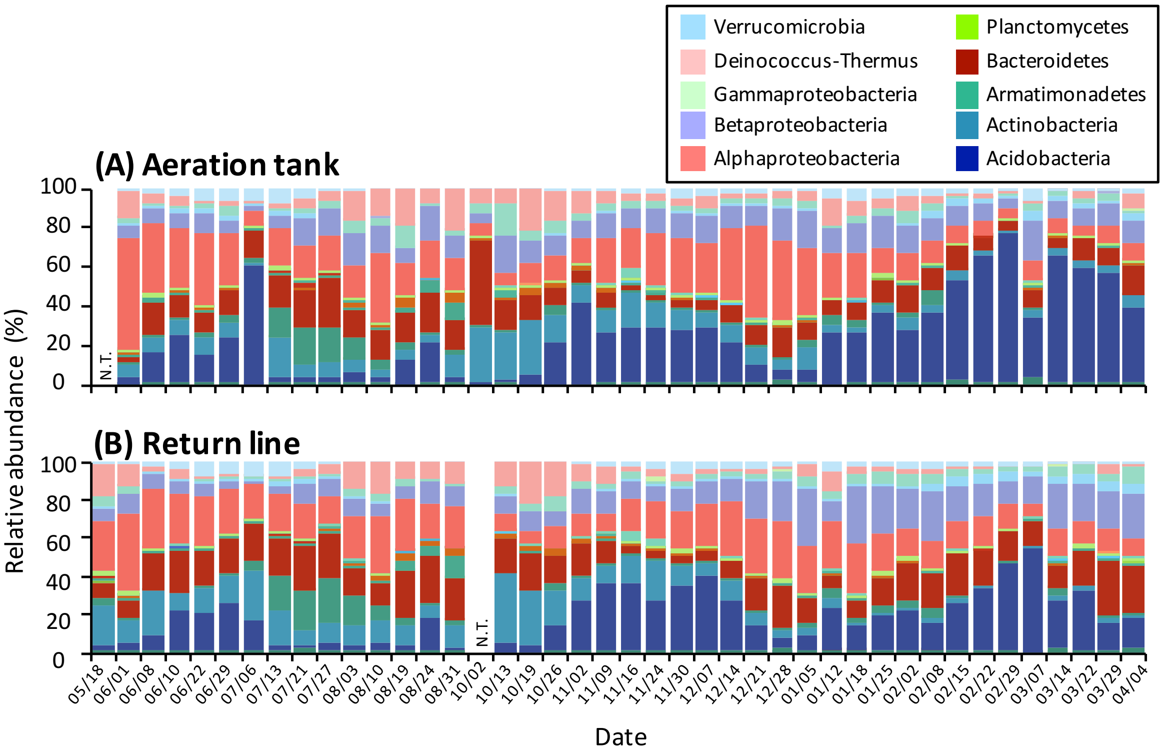

Microbial community structure in the aeration tank (**A**) and return line (**B**) of the full-scale treatment system as determined by Illumina sequencing of 16S rRNA genes. Phylogenetic groups are indicated by colors and their taxonomies are shown at the upper part of the graph. The details of the Illumina sequence libraries are shown in Table S3. N.T. indicates not tested.

Supplementary Figure S8: High-resolution relative abundances of the identified 1,4-dioxane degraders in the full-scale activated sludge system

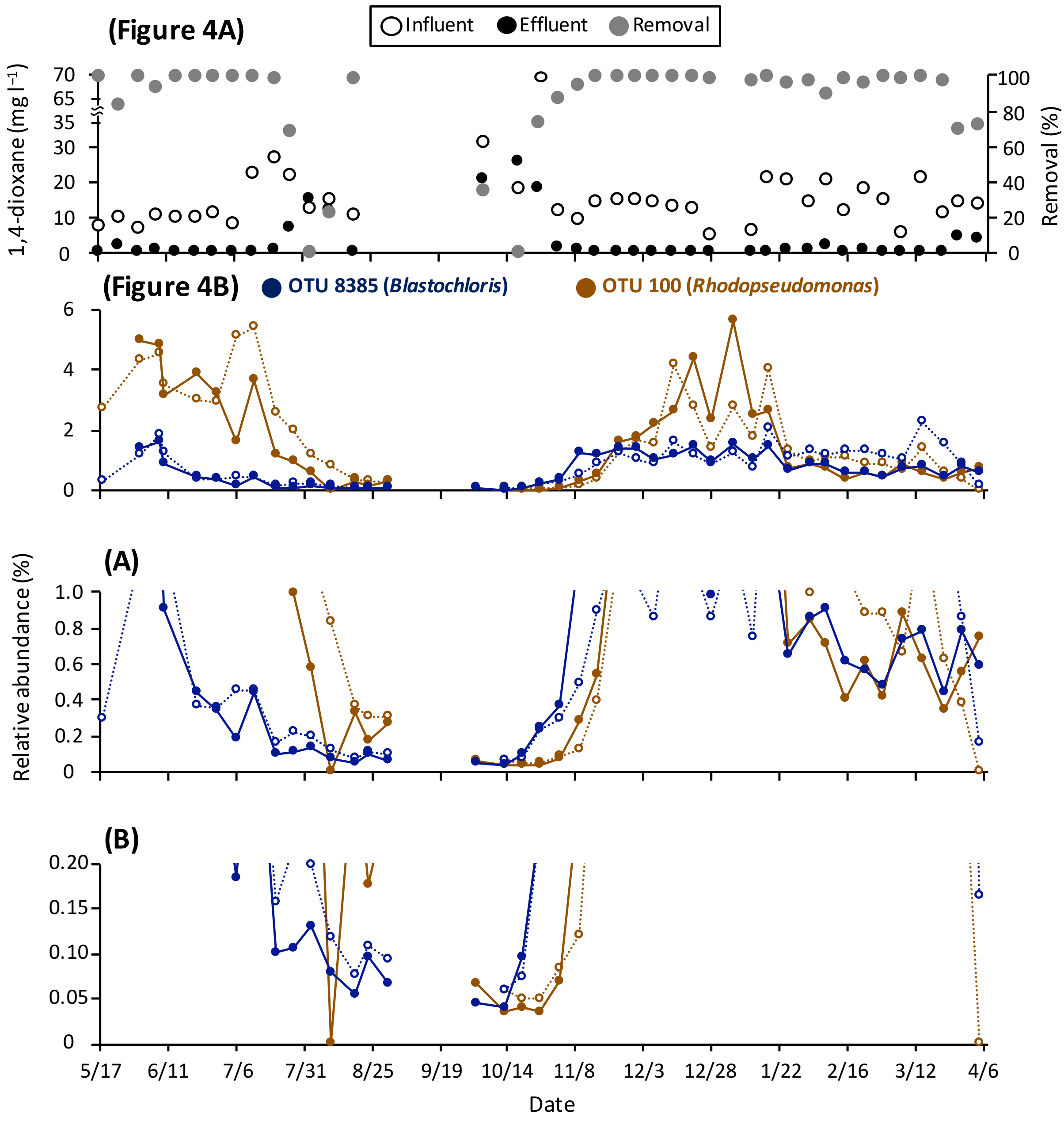

The detailed dynamics of the identified 1,4-dioxane-degrading OTUs 8385, 100 (**A** and **B**), 2230 (**C** and **D**), 12266, and 8474 (**E**) in the aeration tank (closed symbols and solid lines) and return line (open symbols and dotted lines) of the full-scale treatment system. Genera of the closest relatives of the OTUs are indicated in the parentheses of explanatory notes. The relative abundance of each 1,4-dioxane degrader was determined by Illumina sequencing of 16S rRNA genes. The details of the Illumina sequence libraries are summarized in Table S3.

Supplementary Figure S8 (continued)

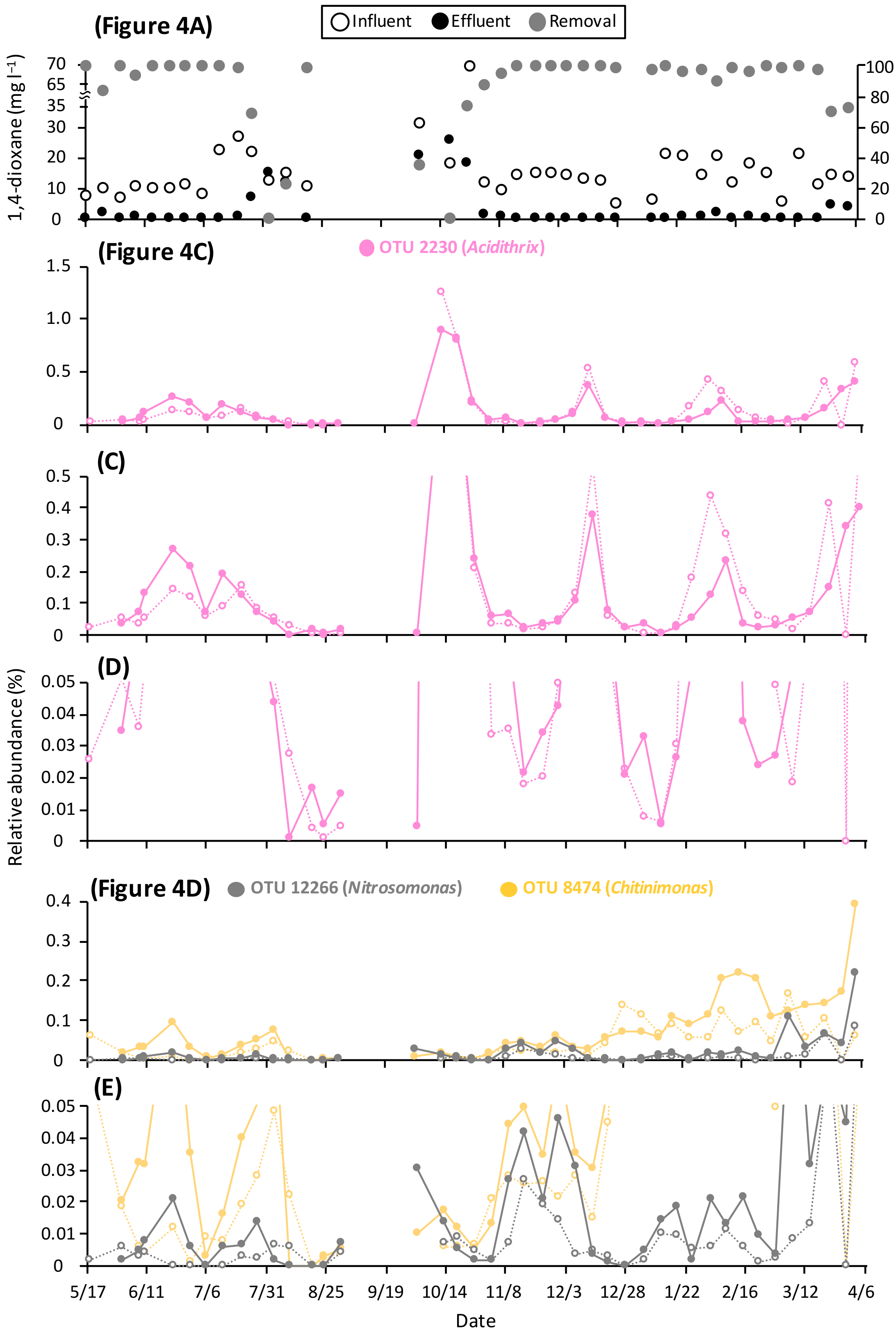

Supplementary Figure S9: Changes in the MLSS (total biomass) and the estimated absolute abundances of the identified 1,4-dioxane degraders in the full-scale system

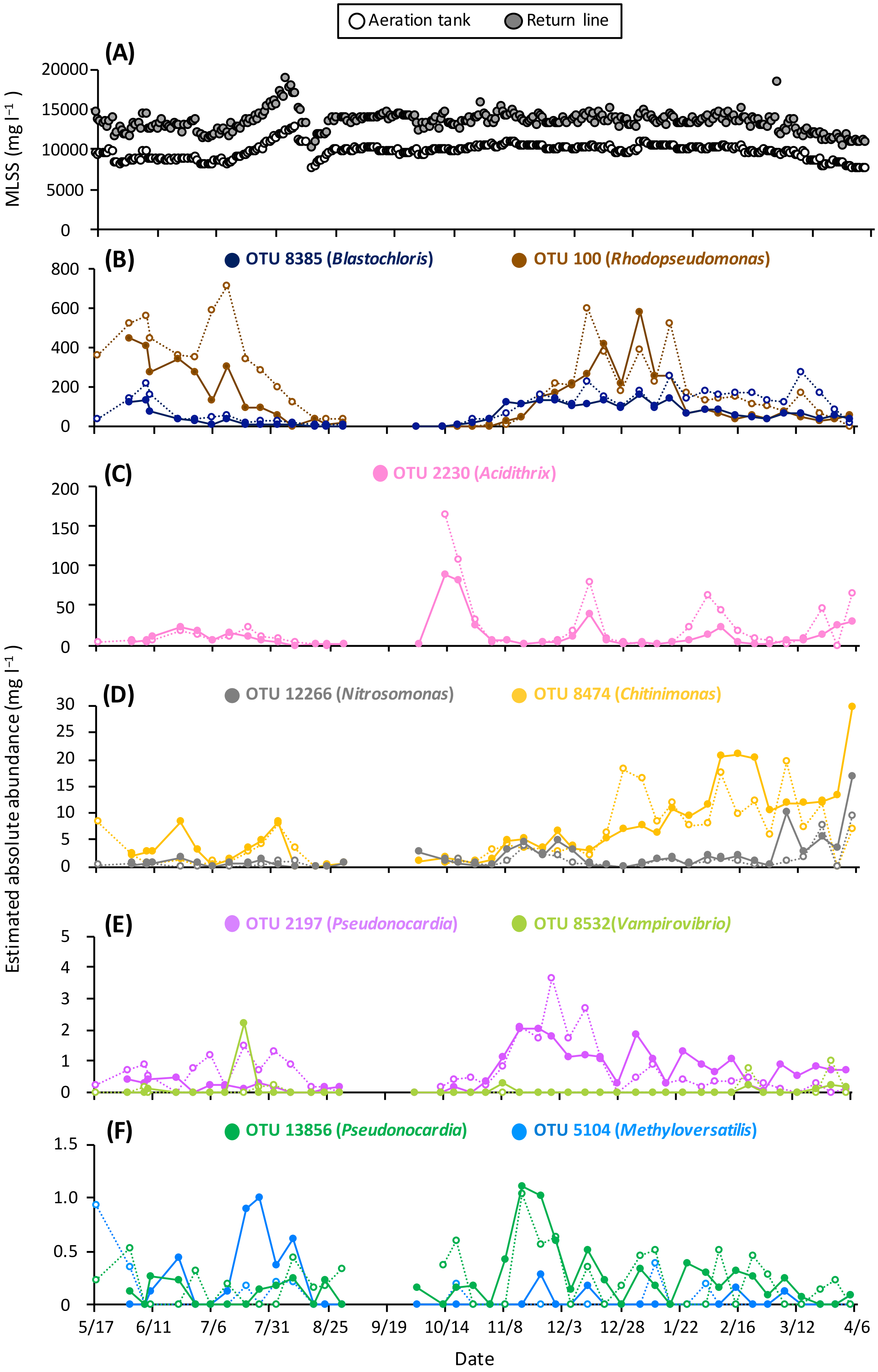

Time course of the mixed liquor suspended solid (MLSS) in the full-scale treatment system (**A**) (open symbols, aeration tank; gray-color symbols, return line). (**B**−**F**) The calculated absolute abundances of the identified 1,4-dioxane degraders in the aeration tank (closed symbols and solid lines) and return line (open symbols and dotted lines) of the full-scale system. Genera of the closest relatives of the OTUs are indicated in the parentheses of explanatory notes. The relative abundance of each 1,4-dioxane degrader was determined by Illumina sequencing of 16S rRNA genes (Figure 4). The details of the Illumina sequence libraries are summarized in Table S3.

Supplementary Table S1: The CsTFA BDs of the selected RNA density fractions and the total sequence number of the Illumina sequence libraries

Treatment	Density fraction [a]			Number of sequences [c]			
	Fraction name [b]	BD (g ml^{-1})	std	average		min	max
^{13}C	1H	1.805	(±0.0014)	57,688	(±3,338)	55,057	61,443
	2H	1.796	(±0.0014)	62,124	(±6,838)	56,351	69,676
	3H	1.790	(±0.0014)	66,246	(±17,406)	54,277	86,214
	L	1.771	(±0.0013)	73,515	(±8,610)	63,655	79,550
Unlabeled	1H	1.806	(±0.0014)	N.A. [d]	N.A.	N.A.	N.A.
	2H	1.799	(±0.0014)	56,161	(±11,009)	42,808	61,897
	3H	1.792	(±0.0014)	60,491	(±3,598)	58,082	64,627
	L	1.771	(±0.0013)	72,409	(±11,500)	60,646	83,626

a The buoyant densities (BDs) of the RNA density fractions (*n*=3) and the number of the sequences analyzed (minimum no., maximum no, *n*=3) are summarized. The CsTFA BDs (g ml^{-1}) were determined from each set of different tubes for isopycnic centrifugation done in triplicate. The standard deviations of three replications are indicated in parentheses.

b Density fractions of RNA are indicated as the 1H (heaviest), 2H (second-heaviest), 3H (third-heaviest) and L (light) fractions.

c 16S rRNA sequences were phylogenetically characterized from each set of the different sequence libraries constructed in triplicate, and the standard deviations of three replications are indicated in parentheses.

d N.A. indicates no amplification product from RT-PCR.

Supplementary Table S2: The total sequence number of 16S rRNA molecules in the Illumina sequence libraries

Treatment	Time (hour)	Number of sequences [a]			
		average		min	max
^{13}C	0	86,662	(±6,379)	79,633	92,083
	2	91,523	(±3,420)	87,580	93,683
	4	110,060	(±17,406)	101,640	110,612
	6	87,720	(±2,385)	85,063	89,676
	8	116,059	(±25,358)	97,810	145,015

a 16S rRNA molecules were phylogenetically characterized from each set of the different sequence libraries constructed in triplicate, and the standard deviations of three replications are indicated in parentheses.

Supplementary Table S3: Sample overview and summary of the Illumina sequence data from the full-scale activated sludge system

Sample	Sampling date	No. of sequences	Alpha-diversity indices [a]						Sample	Sampling date	No. of sequences	Alpha-diversity indices [a]					
			Chao1		Shannon		1/Simpson					Chao1		Shannon		1/Simpson	
	2015/6/1	68,860	4680.0	± 305.2	5.09	± 0.01	8.41	± 0.08		2015/5/18	57,964	6697.5	± 386.7	5.95	± 0.01	15.73	± 0.13
	2015/6/8	81,425	5142.1	± 288.4	5.51	± 0.01	14.37	± 0.08		2015/6/1	70,751	8826.9	± 389.5	6.30	± 0.01	19.46	± 0.14
	2015/6/10	66,678	4971.3	± 262.4	5.44	± 0.02	12.19	± 0.11		2015/6/8	68,946	6185.8	± 360.8	5.47	± 0.02	12.71	± 0.08
	2015/6/22	77,433	7087.5	± 412.7	6.23	± 0.02	19.17	± 0.22		2015/6/10	74,239	5896.3	± 440.4	5.23	± 0.01	10.89	± 0.10
	2015/6/29	65,102	4320.3	± 205.7	5.50	± 0.01	12.84	± 0.10		2015/6/22	59,958	5488.7	± 336.5	5.29	± 0.02	11.93	± 0.10
	2015/7/6	64,914	1779.3	± 167.1	3.12	± 0.02	2.85	± 0.03		2015/6/29	76,748	5476.2	± 286.7	5.08	± 0.03	10.15	± 0.10
	2015/7/13	68,969	3179.1	± 173.5	5.28	± 0.01	13.91	± 0.09		2015/7/6	66,700	4953.4	± 367.2	5.14	± 0.02	11.17	± 0.10
	2015/7/21	74,509	3424.5	± 241.4	5.62	± 0.01	16.56	± 0.16		2015/7/13	65,559	6217.7	± 485.8	5.63	± 0.02	13.41	± 0.10
	2015/7/27	66,946	5381.0	± 252.7	5.57	± 0.02	15.05	± 0.18		2015/7/21	72,839	7633.6	± 321.1	6.04	± 0.01	15.88	± 0.15
	2015/8/3	56,934	4149.8	± 355.7	5.81	± 0.01	19.06	± 0.12		2015/7/27	82,714	5450.4	± 190.6	5.59	± 0.02	13.04	± 0.07
	2015/8/10	93,656	6013.5	± 209.1	5.44	± 0.02	12.18	± 0.13		2015/8/3	76,555	7348.6	± 493.8	5.90	± 0.01	17.76	± 0.16
	2015/8/19	65,488	3333.0	± 231.4	5.44	± 0.01	15.01	± 0.13		2015/8/10	68,447	5885.3	± 307.5	5.63	± 0.01	14.79	± 0.12
	2015/8/24	70,167	5468.5	± 454.8	5.55	± 0.01	14.34	± 0.09		2015/8/19	70,293	4854.1	± 278.3	5.56	± 0.01	18.31	± 0.14
	2015/8/31	58,328	2655.5	± 164.6	5.66	± 0.01	15.82	± 0.19		2015/8/24	78,528	5562.6	± 170.7	5.68	± 0.01	16.22	± 0.12
	2015/10/2	58,773	2432.5	± 195.9	4.86	± 0.01	9.68	± 0.07		2015/8/31	78,738	7303.6	± 277.5	6.23	± 0.01	20.15	± 0.16
	2015/10/13	58,192	6625.2	± 200.2	6.34	± 0.01	22.46	± 0.13		2015/10/13	70,475	6308.6	± 288.5	5.59	± 0.02	12.72	± 0.09
	2015/10/19	59,817	5561.6	± 532.3	5.59	± 0.01	12.84	± 0.06		2015/10/19	66,078	5411.3	± 345.2	5.26	± 0.02	10.71	± 0.11
	2015/10/26	59,835	3301.2	± 261.5	5.23	± 0.01	12.25	± 0.08		2015/10/26	64,694	6618.9	± 318.1	5.78	± 0.01	15.81	± 0.16
	2015/11/2	54,809	2619.9	± 125.8	4.57	± 0.01	5.60	± 0.04		2015/11/2	62,550	6413.3	± 303.6	5.64	± 0.02	11.95	± 0.14
Aeration tank	2015/11/9	74,722	9807.7	± 525.3	6.45	± 0.02	15.31	± 0.16	Return line	2015/11/9	68,058	6340.6	± 428.7	5.43	± 0.02	7.87	± 0.10
	2015/11/16	64,954	7529.8	± 299.6	5.90	± 0.01	11.20	± 0.10		2015/11/16	77,950	8306.8	± 388.7	5.68	± 0.02	8.26	± 0.06
	2015/11/24	67,007	8010.3	± 537.3	6.11	± 0.02	12.56	± 0.13		2015/11/24	68,011	6645.5	± 388.2	5.98	± 0.01	13.84	± 0.11
	2015/11/30	67,440	7946.9	± 247.9	6.16	± 0.02	13.51	± 0.20		2015/11/30	84,293	5886.0	± 209.0	5.59	± 0.01	9.27	± 0.10
	2015/12/7	70,992	8064.1	± 416.4	5.98	± 0.03	12.16	± 0.11		2015/12/7	85,761	7000.6	± 283.5	5.29	± 0.03	6.82	± 0.08
	2015/12/14	58,646	7202.3	± 390.9	5.97	± 0.01	15.55	± 0.14		2015/12/14	80,688	8502.4	± 387.1	6.02	± 0.01	13.30	± 0.16
	2015/12/21	81,145	7635.1	± 324.0	6.21	± 0.02	17.95	± 0.17		2015/12/21	75,713	9361.7	± 564.9	6.31	± 0.01	19.04	± 0.16
	2015/12/28	60,684	8752.0	± 468.2	6.23	± 0.01	15.63	± 0.11		2015/12/28	69,466	7983.2	± 479.2	6.12	± 0.02	15.06	± 0.15
	2016/1/5	60,575	9652.8	± 475.3	7.03	± 0.02	37.21	± 0.34		2016/1/5	61,844	7529.9	± 442.8	6.37	± 0.01	23.30	± 0.19
	2016/1/12	57,242	6109.6	± 313.3	5.49	± 0.02	11.19	± 0.10		2016/1/12	99,807	8094.3	± 367.9	6.25	± 0.02	16.17	± 0.22
	2016/1/18	60,292	7463.1	± 388.8	6.06	± 0.02	14.05	± 0.17		2016/1/18	94,282	9003.6	± 697.3	6.64	± 0.01	25.22	± 0.17
	2016/1/25	53,070	6952.1	± 305.0	5.55	± 0.01	8.18	± 0.06		2016/1/25	90,278	8801.5	± 386.3	6.46	± 0.02	19.59	± 0.32
	2016/2/2	33,496	5834.3	± 136.3	5.82	± 0.02	11.67	± 0.04		2016/2/2	70,019	7484.8	± 211.6	6.17	± 0.02	14.77	± 0.11
	2016/2/8	61,747	7822.3	± 375.8	5.71	± 0.02	8.24	± 0.10		2016/2/8	80,065	9693.3	± 454.0	6.45	± 0.03	19.75	± 0.35
	2016/2/15	60,198	5728.2	± 262.2	4.61	± 0.02	4.15	± 0.03		2016/2/15	70,004	7398.3	± 444.3	5.92	± 0.02	11.50	± 0.13
	2016/2/22	74,410	5229.0	± 293.7	3.76	± 0.02	2.63	± 0.02		2016/2/22	82,360	7326.2	± 264.6	5.50	± 0.02	8.54	± 0.11
	2016/2/29	106,551	4556.7	± 244.7	2.82	± 0.03	1.85	± 0.01		2016/2/29	82,813	6514.6	± 250.3	4.82	± 0.02	5.04	± 0.04
	2016/3/7	73,035	10299.0	± 381.0	6.54	± 0.03	11.47	± 0.16		2016/3/7	96,044	3471.2	± 214.6	3.80	± 0.02	3.42	± 0.03
	2016/3/14	104,898	4833.6	± 398.8	3.74	± 0.02	2.60	± 0.02		2016/3/14	85,174	11131.7	± 295.3	6.60	± 0.02	13.87	± 0.13
	2016/3/22	81,409	4747.1	± 407.9	3.80	± 0.02	3.07	± 0.03		2016/3/22	79,268	6592.1	± 213.2	5.53	± 0.02	8.74	± 0.11
	2016/3/29	87,567	6250.6	± 278.3	4.46	± 0.02	3.61	± 0.04		2016/3/29	92,780	8561.2	± 437.8	6.11	± 0.02	13.52	± 0.17
	2016/4/4	82,325	6084.3	± 387.0	5.18	± 0.02	7.04	± 0.05		2016/4/4	77,266	8758.0	± 264.2	6.78	± 0.01	19.79	± 0.10

a Each alpha-diversity index was calculated based on an equal number (*n*= 28,802) of sequences sub-sampled 10 times from original libraries.

Supplementary Table S4: Summary of the dynamics of 1,4-dioxane degraders in relation to reactor performance in the full-scale treatment system

Date	Dynamics of the physicochemical parameters	Dynamics of the 1,4-dioxane degraders
May 17th to July 6th (except on May 25th and June 8th)	High 1,4-dioxane removal efficiencies (98.7%−99.2%)<br>Nearly constant temperatures at <30.7°C (26.5°C−30.6°C)<br>DO levels at 2.7−6.4 mg l^{-1}	High abundances of OTUs 8385 and 100 (0.184%−5.120%)
May 25th	Low 1,4-dioxane removal efficiency (83.7%)	Increases in the OTUs 8385 and 100
June 8th	Low 1,4-dioxane removal efficiency (93.6%)	Increases in the OTUs 2230 and 8474
July 6th to 21st	High 1,4-dioxane removal efficiencies (98.4%−99.6%)<br>A gradual increase in temperature from 28.0°C to 33.0°C<br>A sudden drop of DO from 4.3 to 1.1 mg l^{-1}	Decreases in the OTUs 100, 8385, and 2230<br>Increases in the OTUs 8474, 8532 and 5104
July 27th to August 10th	Low 1,4-dioxane removal efficiencies (0%−68.2%)<br>Inflow of 1,4-dioxane at 12.5−21.9 mg l^{-1}<br>Influent TOC at 446.9−684.5 mg l^{-1}<br>High temperatures (34.0°C−35.4°C)<br>Low DO levels (0.9−1.7 mg l^{-1})	Decreases in all the OTUs<br>(0.001%−0.079% on August 10th)
August 19th	High 1,4-dioxane removal efficiency (98.0%)<br>Decreased temperature at 30.0°C<br>Increased DO at 5.5 mg l^{-1}<br>Lowered influent TOC of 183.4 mg l^{-1}	Increases in the OTUs 100 and 2230
October 5th to 19th	Low 1,4-dioxane removal efficiencies (0%−34.5%)<br>High DO levels (4.2−6.4 mg l^{-1})	Increase in the OTU 2230 from 0.005% to 0.907%
October 19th to November 16th	Recovery of 1,4-dioxane removal efficiency from 73.8% to 93.8%	Increases in the OTUs 8385, 100, 2197 and 13856
November 16th to February 2nd	High 1,4-dioxane removal efficiencies (95.4%−99.4%)<br>Nearly constant temperatures (21.3°C−30.2°C)<br>Sustained DO levels (3.7−5.9 mg l^{-1})	Increases in the OTUs 100 and 8474<br>Maintenance at a constant level of the OTU 8385<br>Fluctuations for the OTUs 2197 and 13856<br>Transient appearances for the OTUs 2230 and 12266
February 8th	Low 1,4-dioxane removal efficiency (89.2%)	Increases in the OTUs 2230, 8474 and 12266
February 15th to March 22nd	High 1,4-dioxane removal efficiencies (95.8%−99.6%)	Increases in the OTUs 2230, 8474 and 12266
